# Reduced miR-184-3p expression protects pancreatic β-cells from lipotoxic and proinflammatory apoptosis in type 2 diabetes via CRTC1 upregulation

**DOI:** 10.1038/s41420-022-01142-x

**Published:** 2022-07-29

**Authors:** Giuseppina E. Grieco, Noemi Brusco, Daniela Fignani, Laura Nigi, Caterina Formichi, Giada Licata, Lorella Marselli, Piero Marchetti, Laura Salvini, Laura Tinti, Agnese Po, Elisabetta Ferretti, Guido Sebastiani, Francesco Dotta

**Affiliations:** 1 Diabetes Unit, Department of Medicine, Surgery and Neurosciences, University of Siena, Fondazione Umberto Di Mario ONLUS c/o Toscana Life Science, Siena, Italy; 2 Department of Clinical and Experimental Medicine, Islet Cell Laboratory, University of Pisa, Pisa, Italy; 3 TLS-Toscana Life Sciences Foundation, Siena, Italy; 4 Department of Experimental Medicine, Sapienza University, 00161 Rome, Italy; 5 Tuscany Centre for Precision Medicine (CReMeP), Siena, Italy

**Keywords:** Mechanisms of disease, Type 2 diabetes

## Abstract

The loss of functional β-cell mass in type 2 diabetes (T2D) is associated with molecular events that include β-cell apoptosis, dysfunction and/or dedifferentiation. MicroRNA miR-184-3p has been shown to be involved in several β-cell functions, including insulin secretion, proliferation and survival. However, the downstream targets and upstream regulators of miR-184-3p have not been fully elucidated. Here, we show reduced miR-184-3p levels in human T2D pancreatic islets, whereas its direct target CREB regulated transcription coactivator 1 (CRTC1) was increased and protects β-cells from lipotoxicity- and inflammation-induced apoptosis. Downregulation of miR-184-3p in β-cells leads to upregulation of CRTC1 at both the mRNA and protein levels. Remarkably, the protective effect of miR-184-3p is dependent on CRTC1, as its silencing in human β-cells abrogates the protective mechanism mediated by inhibition of miR-184-3p. Furthermore, in accordance with miR-184-3p downregulation, we also found that the β-cell-specific transcription factor NKX6.1, DNA-binding sites of which are predicted in the promoter sequence of human and mouse MIR184 gene, is reduced in human pancreatic T2D islets. Using chromatin immunoprecipitation analysis and mRNA silencing experiments, we demonstrated that NKX6.1 directly controls both human and murine miR-184 expression. In summary, we provide evidence that the decrease in NKX6.1 expression is accompanied by a significant reduction in miR-184-3p expression and that reduction of miR-184-3p protects β-cells from apoptosis through a CRTC1-dependent mechanism.

## Introduction

Type 2 diabetes mellitus (T2D) is a metabolic disease caused by the interaction of genetic and environmental factors. Exposure to gluco-lipotoxic and inflammatory stress progressively leads to β-cell failure, resulting in a significant reduction in functional β-cell mass and alteration of glucose homeostasis, which in turn leads to chronic hyperglycaemia [1–3]. Although it has been shown that β-cell mass is markedly reduced in T2D patients due to the apoptotic effects of gluco-lipotoxic stressors and inflammatory mediators [1–3], it has recently been reported that such phenomenon may be overestimated, suggesting the existence of additional mechanisms involved in the pathogenesis of T2D [4]. Several studies reported that dedifferentiation of β-cells may occur in T2D, thus actively contributing to their dysfunction and reduction of functional β-cell mass [5, 6]. Interestingly, recent evidence suggest that dedifferentiation is associated with the acquisition of a protected phenotype that makes β-cells more resistant to metabolic and/or inflammatory insults, despite being dysfunctional [5, 7]. These findings are reinforced by the notion that β-cell failure can be seemingly reversed [8–10], suggesting that a subset of β-cells is dysfunctional but ready to potentially resume function [10–12].

MicroRNAs (miRNAs), a class of small endogenous RNAs that regulate gene expression [13, 14], have been reported to be pivotal modulators and rheostats of cell differentiation [15–17], survival [18], phenotype maintenance [19] and function [20, 21]. Among these, miR-184-3p is one of the most enriched miRNAs in pancreatic islets and in β-cells [22]. Previous studies showed a reduced expression of miR-184-3p in pancreatic islets from T2D murine models (e.g., db/db and ob/ob mice) [23] as well as in human pancreatic islets from T2D donors [22]. Several lines of evidence suggest a possible role of miR-184-3p in the protective mechanisms of β-cells during metabolic insults typical of T2D [22]. Tattikota et al. demonstrated that downregulation of miR-184-3p modulates Ago2 (Argonaute 2) expression, leading to compensatory β-cell expansion through indirect modulation of miR-375 activity [22]. Furthermore, downregulation of miR-184-3p has been reported to protect the mouse β-cell line MIN6 from metabolic and pro-inflammatory cell stress, suggesting an anti-apoptotic role of miR-184-3p [23, 24]. In addition, miR-184-3p regulates insulin secretion through direct targeting of solute carrier family 25 member 22 (Slc25A2), a mitochondrial glutamate carrier involved in glutamate transport across the inner mitochondrial membrane [25]. Finally, although another report [26] has shown that miR-184-3p is also regulated by the glucose sensor AMPK, the exact molecular mechanisms by which miR-184-3p protects β-cells have not yet been fully elucidated.

Here, we provide further evidence that miR-184-3p is downregulated in human T2D pancreatic islets and show that its reduction is due to the loss of NKX6.1, a β-cell phenotype-associated transcription factor. Furthermore, we show that low miR-184-3p levels protect β-cells from metabolic/inflammatory stress by regulating the transcription factor CREB transcriptional coactivator 1 (CRTC1).

## Results

### Downregulation of miR-184-3p in human type 2 diabetic (T2D) pancreatic islets protects β-cells from apoptosis

**Fig. 1:**
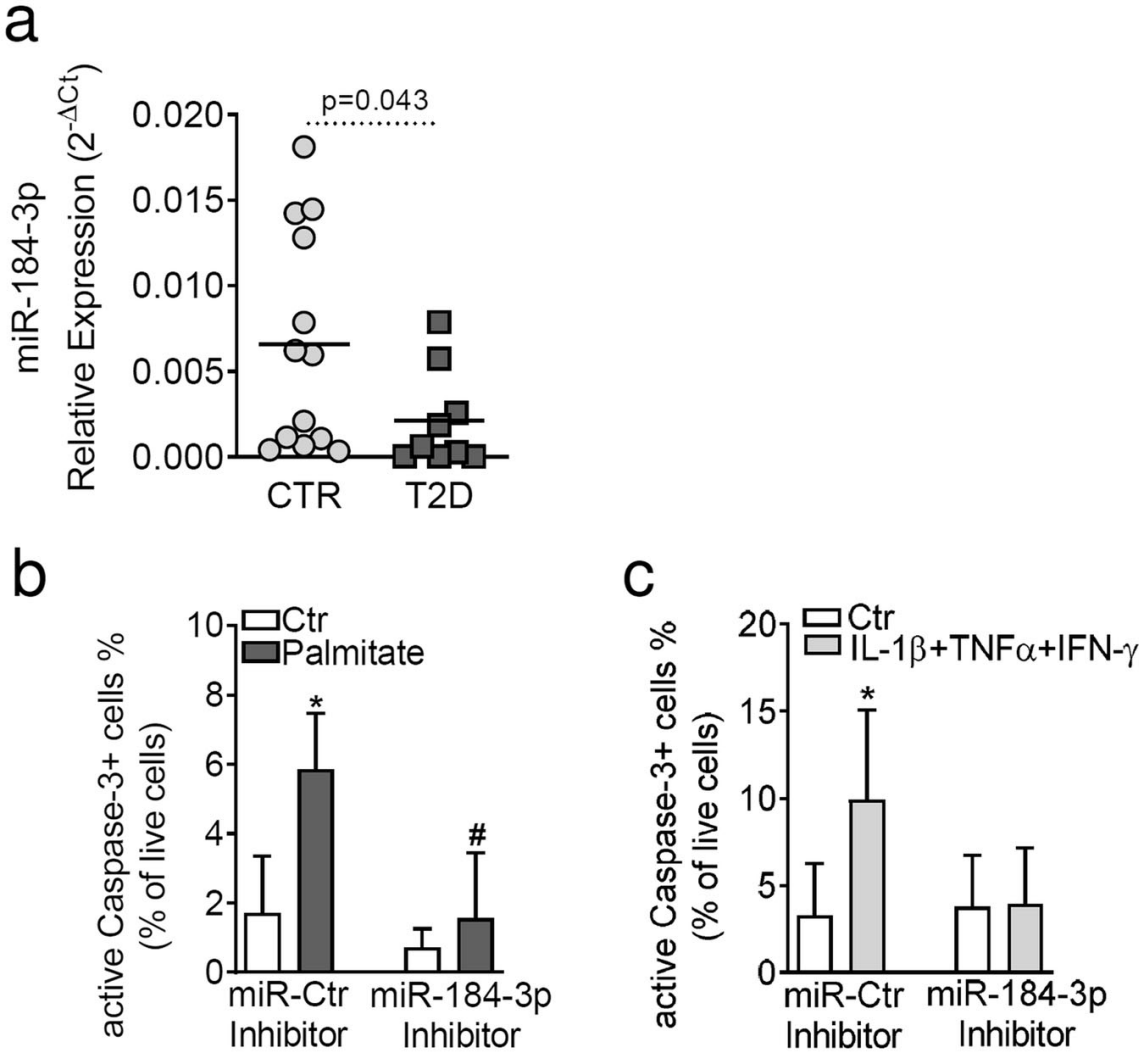
miR-184-3p is downregulated in human pancreatic islets in T2D and protects β-cells from palmitate- and cytokine-induced apoptosis. **a** RT-Real-time PCR analysis of miR-184-3p expression in human T2D pancreatic islets (*n* = 9; dark grey squares) and in non-diabetic (*n* = 13; light grey circles) multi-organ donors; data are presented as 2^−ΔCt^ relative expression single point values normalised with RNU44 and RNU48, along with the mean; statistics with Mann–Whitney *U* test. **b** Cytofluorimetric evaluation of cleaved Caspase-3 positive (cleaved-CASP-3^+^) EndoC-βH1 cells each after 48 h palmitate treatment (*p = 0.0008 vs. untreated miR-Ctr inhibitor; ^#^*p* = 0.0005 vs. Palmitate-treated miR-Ctr inhibitor transfected) and **c** after 24 h cytokine mix (IL -1β+TNF-α+IFN-γ) treatment (*p = 0.032 vs. untreated miR-Ctr inhibitor). Data are presented as mean ± SD of the percentage (%) of positive cells to total live cells. Statistics were performed using ANOVA analysis with Bonferroni’s multiple comparison test (*n* = 4 independent experiments).

MiR-184-3p has been shown to play a central role in fine-tuning and modulating pancreatic β-cell function. To further decipher the role of miR-184-3p, we first analysed its expression in human pancreatic islets isolated from the pancreas of *n* = 13 non-diabetic and *n* = 9 T2D multi-organ donors (Supplementary Table 1). We found a significant decrease in miR-184-3p expression in pancreatic islets isolated from T2D compared to non-diabetic donors (–2.9-fold change compared to CTR, *p* = 0.043 non-parametric Mann–Whitney *U* test) (Fig. 1a). No correlation was found between donors age or BMI and miR-184-3p expression levels (data not shown).

In pancreatic islets from T2D mouse models (*db/db* and *ob/ob*) and in mouse β-cell line (MIN6), the reduction in miR-184-3p expression has been shown to be involved in a pro-survival process that may leverage a protective mechanism that has not yet been elucidated [23]. To test whether such protection also occurs in human β-cells, we inhibited miR-184-3p in the human β-cell line EndoC-βH1 [27] exposed or not to palmitate or a cytokine mix (IL-1β+TNF-α+IFN-γ) to simulate lipotoxic or inflammatory stress. Inhibition of miR-184-3p protected human β-cells from apoptosis after 48 h of palmitate exposure, as shown by cleaved Caspase-3 staining (Fig. 1b and Supplementary Fig. 1a) and confirmed by counting pyknotic nuclei (Supplementary Fig. 1b). Such protection was also observed when EndoC-βH1 was exposed to inflammatory stimuli using a mix of pro-inflammatory cytokines (IL-1β+TNF-α+IFN-γ) (Fig. 1c and Supplementary Fig. 1c, d), demonstrating that inhibition of miR-184-3p protects β-cells from both metabolic and inflammatory stress conditions. Overall, these data confirm that miR-184-3p expression is reduced in pancreatic islets derived from T2D multi-organ donors and that this molecular feature is involved in the protection of β-cells from palmitate- and cytokine-induced apoptosis.

### The predicted target of miR-184-3p, CREB transcriptional coactivator 1 (CRTC1), is upregulated in human LCM-captured β-cells and in pancreatic islets from type 2 diabetic donors

**Fig. 2:**
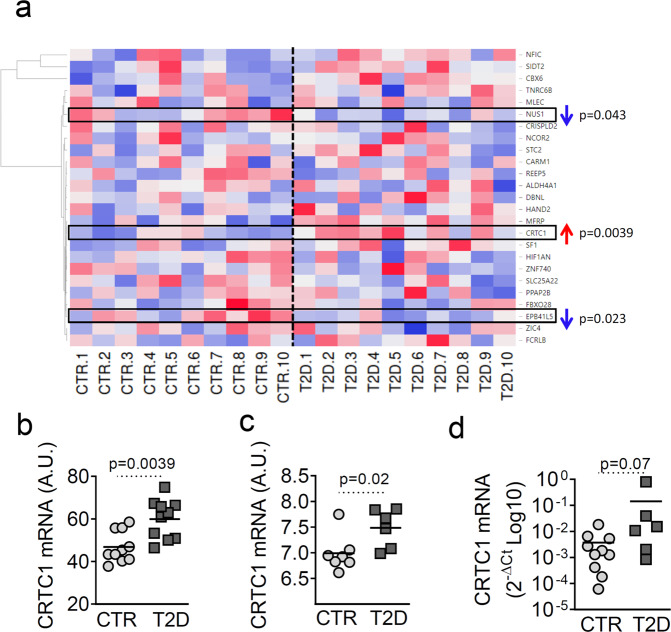
CRTC1 is upregulated in LCM-captured human β-cells and in pancreatic islets from T2D donors. **a** Hierarchical clustering heatmap expression analysis of hsa-miR-184-3p predicted target genes (*n* = 25) detected in LCM β-cells from *n* = 10 T2D vs. *n* = 10 non-diabetic donors (data previously published and deposited in GEO dataset GDS3782-GSE20966). Gene expression values are shown as arbitrary unit count (A.U.) values on a colour scale (high expression: red; low expression: blue). **b** Dot-plot diagram showing the CRTC1 expression values analysed in the dataset reported in **a**. The values are reported as arbitrary units (A.U.) along with the mean. **c** Dot plot showing CRTC1 expression in isolated human pancreatic islets from *n* = 6 T2D compared to *n* = 7 non-diabetic donors (data previously published and included in GEO dataset GDS3882-GSE25724). Values are reported as A.U. along with the mean. **d** RT-Real-time PCR validation of CRTC1 expression in human pancreatic islets isolated from *n* = 6 T2D vs. *n* = 10 non-diabetic multi-organ donors; data are reported as 2^−ΔCt^ relative expression single point values normalised by β-ACT and GAPDH; statistics were performed using the Mann–Whitney *U* test (*p* < 0.05).

To further elucidate the function of miR-184-3p, we examined its predicted target genes using the TargetScan 7.1 algorithm (http://www.targetscan.org/vert_71/). We identified 29 high-ranking and conserved predicted target genes (listed in Supplementary Table 2). Since downregulation of miR-184-3p should lead to increased expression of its target genes, we examined a previously published and online dataset of high-throughput gene expression data from laser capture microdissects (LCM) of non-diabetic and T2D human β-cells (Gene Expression Omnibus (GEO) dataset: GDS3782-GSE20966) [28], for expression of miR-184-3p predicted target genes. A total of 25/29 genes were detected in the analysed dataset, with 3/25 showing significantly different expression (Fig. 2a and Supplementary File 1). Among them, CRTC1 was upregulated in T2D compared to non-diabetic LCM β-cells (Fig. 2b) (CRTC1, *p* = 0.0039 Mann–Whitney *U* test), consistent with reduced miR-184-3p expression. Similar differential expression (*p* = 0.02 Mann–Whitney *U* test) was observed in another dataset (GEO dataset: GDS3882-GSE25724) containing genes expression data from non-diabetic and T2D collagenase-isolated human pancreatic islets (Fig. 2c). Finally, we examined CRTC1 mRNA expression by RT-Real-time PCR in a subset of isolated islet samples from non-diabetic and T2D multi-organ donors (Supplementary Table 1). The analysis showed a similar trend (*p* = 0.07 Mann–Whitney *U* test) to that observed in previous datasets (Fig. 2d). These data suggest a possible role of a miR-184-CRTC1 regulatory axis in β-cell survival.

### MiR-184-3p inhibition upregulates CRTC1 expression

**Fig. 3:**
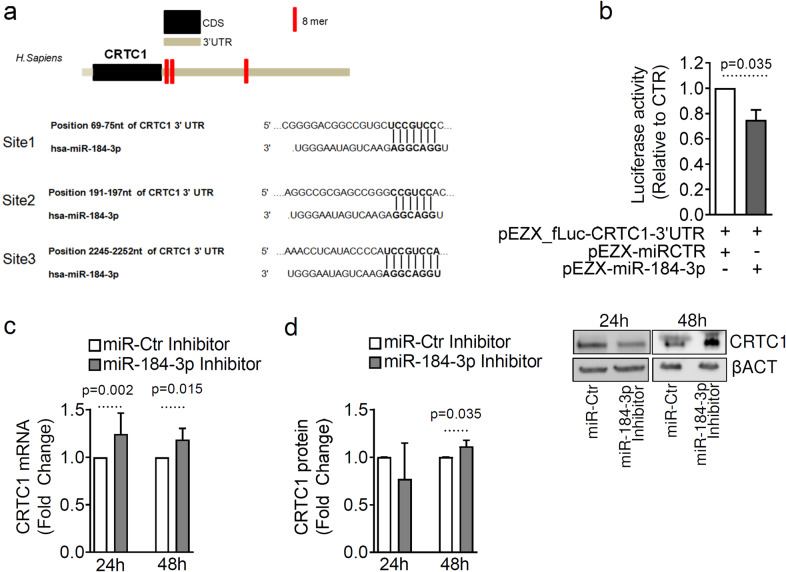
miR-184-3p directly modulates the expression of CRTC1. **a** Top panel: graphical representation of CRTC1 mRNA sequence (ENST00000338797.6) showing 3’UTR and predicted hsa-miR-184-3p binding sites (in red); bottom panel: nucleotide position of hsa-miR-184-3p binding sites and sequence context within the 3’UTR region of CRTC1 mRNA. **b** Luciferase assay in HeLa cells transfected with a plasmid overexpressing hsa-miR-184-3p compared to cells transfected with a scrambled vector; values are given as mean ± SD of relative luciferase activity compared to control, after normalisation of firefly luciferase unit to renilla luciferase activity; statistics using Mann–Whitney *U* test (*n* = 4 independent experiments). **c** RT-Real-time PCR analysis of CRTC1 expression in EndoC-βH1 cells transfected with a synthetic inhibitor of hsa-miR-184-3p; data are expressed as fold change compared to scrambled control; Mann–Whitney *U* test statistics (*n* = 6 independent experiments). **d** Western blot analysis of CRTC1 (78 kDa) in EndoC-βH1 cells transfected with a synthetic inhibitor of hsa-miR-184-3p. Data are presented as mean ± SD of fold-change values of normalised CRTC1/β-Act ratio of miR-184-p inhibitor vs. miR-Ctr inhibitor-transfected samples; statistics using paired Student’s *t*-test (*n* = 6 independent experiments).

Considering that previous reports attribute a critical role to CRTCs in regulating cell metabolism and survival through multiple signalling pathways [29, 30], we then aimed to determine whether CRTC1 is a direct miR-184-3p target gene. The human CRTC1 3’UTR sequence showed 3 binding sites for miR-184-3p (Fig. 3a). Although these binding sites are largely conserved among primates, there is no binding site for miR-184-3p in the mouse CRTC1 3’UTR sequence, despite the high homology between the human and mouse CRTC1 genes sequence (>85%). To assess the binding of miR-184-3p to the 3’UTR of human CRTC1, we first co-transfected the HeLa cell line with a plasmid containing the entire 3’UTR sequence of CRTC1 in a Firefly-Renilla luciferase vector, along with a plasmid expressing miR-184-3p. The luciferase assay showed that overexpression of miR-184-3p resulted in a significant decrease in Firefly luciferase activity, confirming the interaction of miR-184-3p with the 3’UTR sequence of CRTC1 (Fig. 3b). To assess the modulation of CRTC1 expression mediated by miR-184-3p at the mRNA and protein levels, we inhibited miR-184-3p for 24 or 48 h in EndoC-βH1 and in 1.1B4 human β-cell lines as well as in HeLa cells, and then measured CRTC1 expression by RT-Real-time PCR and western blot. Inhibition of miR-184-3p resulted in a significant increase in CRTC1 mRNA expression at 24 and 48 h after transfection and a corresponding CRTC1 protein increase at 48 h in both EndoC-βH1 (Fig. 3c, d and Supplementary Material Original WB) and in the 1.1B4 β-cell line (Supplementary Fig. 2a, b and Supplementary Material Original WB). In HeLa cells, we observed an increase in CRTC1 protein as early as 24 h after transfection (Supplementary Fig. 2c, Fig. 2d and Supplementary Material Original WB).

Overall, these data suggest that miR-184-3p directly regulates CRTC1 expression in the human β-cell line EndoC-βH1 as well as in other contexts.

### CRTC1 upregulation protects β-cells from lipotoxic- and pro-inflammatory-mediated apoptosis

**Fig. 4:**
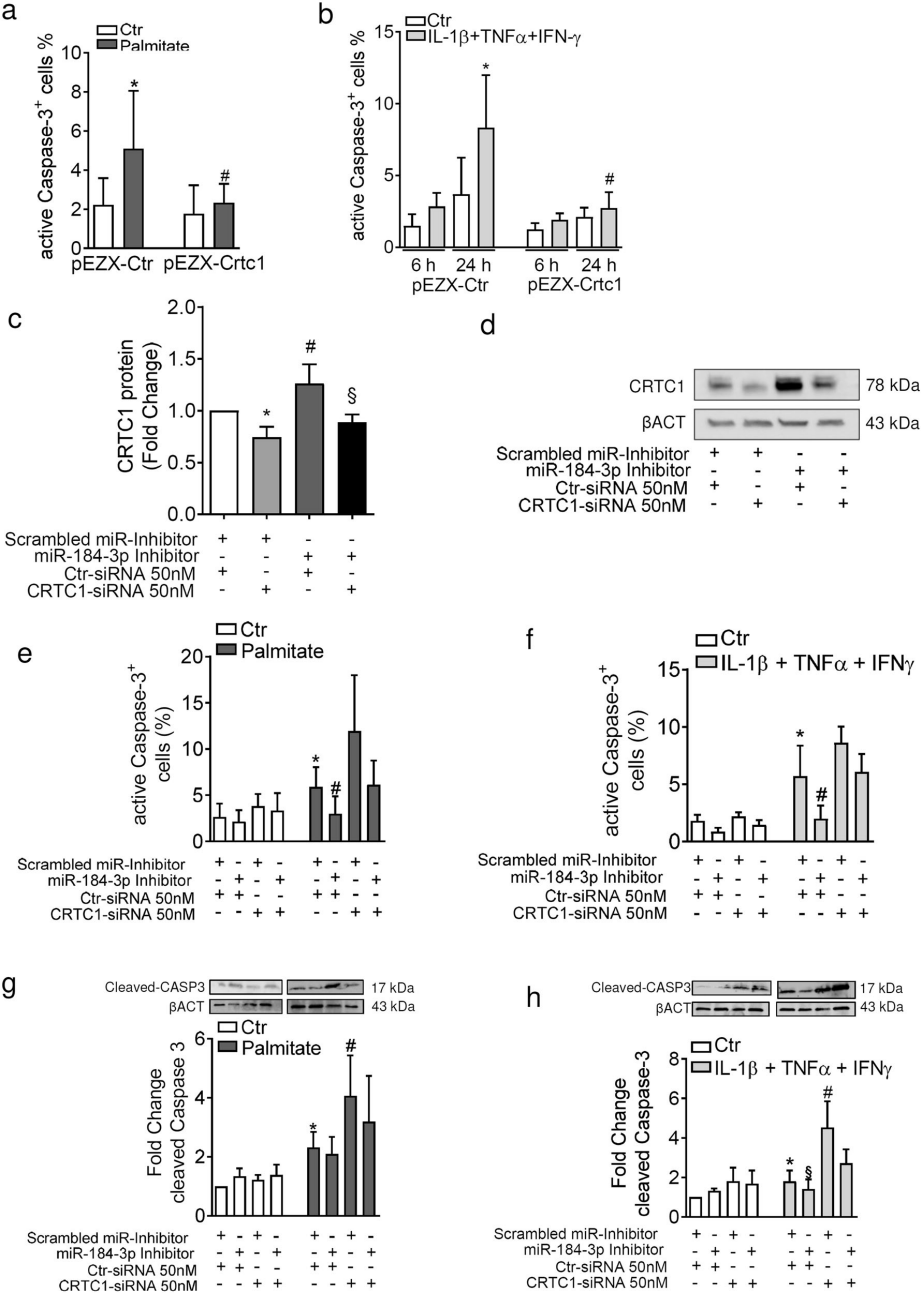
miR-184-3p-mediated protection from palmitate and cytokines-induced apoptosis is dependent on CRTC1 upregulation. **a**, **b** Cytofluorimetric assessment of cleaved Caspase-3 positive MIN6 cells overexpressing or not CRTC1 after 24 h treatment with palmitate **a** (*p < 0.0001 vs. untreated pEZX-Ctr transfected; ^#^*p* < 0.0001 vs. palmitate-treated pEZX-Ctr transfected) or cytokine mix (IL -1β+TNF-α+IFN-γ) treatment for 6 and 24 h **b** (*p = 0.0266 vs. 24 h untreated pEZX-Ctr transfected; ^#^*p* = 0.0359 vs. 24 h cytokine-treated pEZX-Ctr transfected; values are expressed as mean percentage ± SD of Caspase-3^+^ cells to total live cells; statistics were performed using one-way ANOVA with Bonferroni’s multiple comparison test (*n* = 6 independent experiments). **c** Western blot densitometric image analysis of CRTC1 normalised using housekeeping control β-ACT in EndoC-βH1 cells transfected with CRTC1 siRNA (50 nM) in combination or not with miR-184-3p inhibitor (*p = 0.044 vs. scrambled miR inhibitor + Ctr siRNA transfected; ^#^*p* = 0.040 vs. scrambled miR inhibitor + Ctr siRNA transfected; ^§^*p* = 0.0002 vs. scrambled miR inhibitor + CRTC1 siRNA transfected). Data are presented as mean ± SD of fold-change values of normalised CRTC1/β-ACT ratio; statistics were performed using one-way ANOVA with Bonferroni’s multiple comparison test (*n* = 6 independent experiments). **d** Representative western blot of CRTC1 (78 kDa) protein expression and β-ACT (43 kDa) related to the densitometric analysis in (**c**). **e** Cytofluorimetric assessment of cleaved Caspase-3 positive EndoC-βH1 cells transfected with CRTC1 siRNA (50 nM) in combination or not with inhibitor of hsa-miR-184-3p and treated with palmitate for 24 h (*p = 0.02 vs. not treated and scrambled miR inhibitor + Ctr siRNA transfected; ^#^*p* < 0.0001 vs. treated miR-184 inhibitor + Ctr siRNA transfected) or **f** treated with cytokines for 24 h (*p < 0.0001 vs. not treated scrambled miR inhibitor + Ctr siRNA transfected; ^#^*p* = 0.0005 vs. treated and scrambled miR inhibitor + Ctr siRNA transfected). **g** Cleaved Caspase-3 western blot analysis of EndoC-βH1 cells transfected with CRTC1 siRNA (50 nM) in combination or not with inhibitor of hsa-miR-184-3p and treated with palmitate for 24 h (*p = 0.01 vs. not treated scrambled miR inhibitor + Ctr siRNA transfected; ^#^*p* = 0.028 vs. treated and scrambled miR inhibitor + Ctr siRNA transfected). **h** Cleaved Caspase-3 western blot analysis of EndoC-βH1 cells transfected as reported above and treated with cytokines (*p = 0.06 vs. not treated scrambled miR inhibitor + Ctr siRNA transfected; ^§^*p* = 0.022 vs. treated scrambled miR inhibitor + CTR siRNA; ^#^*p* = 0.027 vs. treated and scrambled miR inhibitor + Ctr siRNA transfected). Data are presented as mean ± SD of fold change of normalised cleaved Caspase-3 results with respect to not treated scrambled miR inhibitor + CTR siRNA transfected samples. Statistics were performed using one-way ANOVA with Bonferroni’s multiple comparison test or with paired Student’s *t*-test (*n* = 4–6 independent experiments).

To verify the anti-apoptotic effect of CRTC1, we overexpressed it in MIN6 cells and exposed them to palmitate or to a mix of pro-inflammatory cytokines. As shown, CRTC1 overexpression (Supplementary Fig. 3a, b) protects MIN6 β-cells from palmitate- (Fig. 4a and Supplementary Fig. 4a, b) and from cytokine-induced apoptosis (Fig. 4b and Supplementary Fig. 4c, d).

Importantly, the direct contribution of CRTC1 to miR-184-3p-mediated protection was confirmed in the human β-cell line EndoC-βH1. To test whether CRTC1 plays a prominent role in protecting human β-cell through reduced miR-184-3p expression, CRTC1 was knocked down during treatment with palmitate or cytokines, alone or in combination with inhibition of miR-184-3p. First, to confirm the modulation of CRTC1 protein expression following siRNA or miR-184-3p inhibition, we measured it using western blot and confirmed the results using the targeted mass spectrometry (MS) approach for quantification. Specifically, as expected, both the western blot data (Fig. 4c, d and Supplementary Material Original WB) and MS (Supplementary Fig. 5a–c) analyses showed that: (i) CRTC1-specific siRNA triggers CRTC1 downregulation; (ii) inhibition of miR-184-3p leads to CRTC1 upregulation; (iii) silencing of CRTC1 counterbalances its upregulation mediated by miR-184-3p inhibitor. As expected, the rate of apoptosis, as measured by staining of cleaved Caspase-3 (evaluated both in FACS and western blot analyses), was reduced by inhibition of miR-184-3p in both palmitate and cytokine-exposed EndoC-βH1 cells. Interestingly, silencing of CRTC1 significantly increased the rate of apoptosis upon stress-induction by palmitate and completely abolished the protective effects of miR-184-3p inhibition (Fig. 4e, g, Supplementary Fig. 6a, b and Supplementary Material Original WB). A similar pattern was also observed in cytokine-treated EndoC-βH1 cells (Fig. 4f, h, Supplementary Fig. 7a, b and Supplementary Material Original WB).

Taken together, these data indicate that miR-184-3p-mediated protection of β-cells from apoptosis is exerted mainly by CRTC1.

### β-cell-specific transcription factor NKX6.1 regulates miR-184-3p expression

**Fig. 5:**
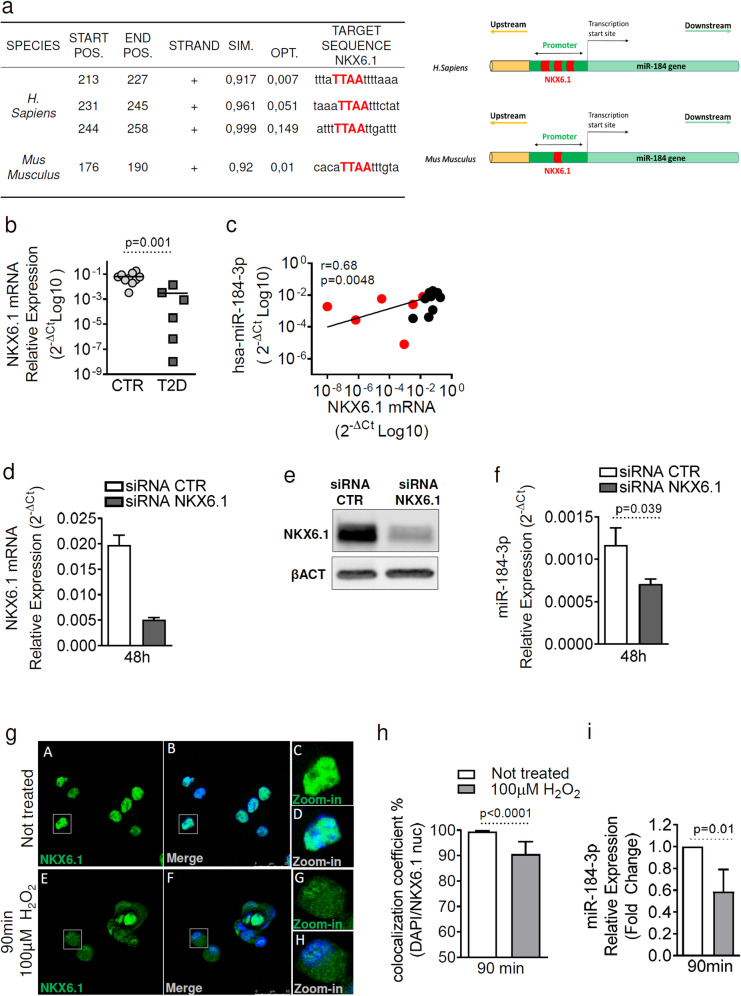
NKX6.1 regulates human and murine miR-184-3p expression. **a** Left panel: table showing predicted binding sites of NKX6.1 in the human and mouse miR-184 gene promoter using MatInspector algorithm. For each predicted binding site, the following parameters are given: species, start/end nucleotide (Start Pos, End Pos) of the TF matrix (with respect to –500 bp of miR-184 TSS, see Sequence in Supplementary material), sequence strand, matrix similarity (SIM.), optimised matrix similarity threshold (OPT.) and target sequence (see Supplementary File 2). Right panel: representative schematic showing human and mouse MIR184 gene promoter and NKX6.1 binding sites (in red). **b** RT-Real-time PCR expression analysis of NKX6.1 in human pancreatic islets with T2D (*n* = 6; dark grey squares) compared to non-diabetic donors (*n* = 10; light grey circles); data are shown as 2^−ΔCt^ relative expression; statistics with Mann–Whitney *U* test. **c** Correlation analysis between expression of NKX6.1 and miR-184-3p in human pancreatic islets from T2D and non-diabetic donors (*n* = 16). Values are expressed as 2^−ΔCt^ (log10) relative expression; *p* value and *r* value by Spearman *R* correlation test. **d**, **e** 48 h silencing of NKX6.1 with siRNA (25 nM) in EndoC-βH1 cell line showed reduction in mRNA (**d**) and protein (**e**) expression; statistics by Mann–Whitney *U* test (*n* = 4). **f** RT-Real-time PCR of miR-184-3p expression in EndoC-βH1 after 48 h NKX6.1 siRNA transfection; data are expressed as mean ± SD of 2^−ΔCt^ normalised values; statistics with Mann–Whitney *U* test (*n* = 4 independent experiments). **g** Immunofluorescence analysis of Nkx6.1 (green) and nuclei (DAPI, blue) in MIN6 cells treated or not with 100 μM H2O2; panel **A** (Nkx6.1) to panel **D** show MIN6 cells not treated; panel **E** to panel **H** show MIN6 cells treated with H_2_O_2_; scale bar = 25 µm. **h** Analysis of colocalisation coefficient for quantification of NKX6.1 and nuclear staining with or without treatment with 100 μM of H_2_O_2_. Data are expressed as mean ± SD of the percentage of NKX6.1 signal overlapping nuclear staining with DAPI. Statistics with Mann–Whitney *U* test (*n* = 5 independent experiments). **i** RT-Real-time PCR analysis of miR-184-3p expression in MIN6 cells after 100 μM of H_2_O_2_ treatment. Data are presented as mean ± SD of fold change of MIN6 cells treated with H_2_O_2_ compared to untreated samples; statistics with Mann–Whitney *U* test (*n* = 5 independent experiments).

The functions of miR-184-3p have been partially elucidated, neverthless the factors regulating transcription and expression of the MIR184 gene in β-cells have not yet been identified. Therefore, we analysed the proximal MIR184 gene promoter sequence (0.5 kb upstream of the MIR184 transcription start site [31]) to identify putative factors regulating its transcription (Supplementary Fig. 8). In silico analysis of the human MIR184 gene promoter revealed 234 predicted binding sites for transcription factors. Among these, we identified 3 binding sites for the β-cell-specific transcription factor NKX6.1, while only a single binding site was detected in the murine miR-184-3p promoter sequence (Fig. 5a, left and right panels, and Supplementary File 2). NKX6.1 DNA-responsive elements (DRE) were characterised by the typical homeodomain core sequence binding motif belonging to NKX6.1 (NNTTAANN) [32] (Fig. 5a, left panel). Given the fundamental role of NKX6.1 in pancreatic endocrine cell differentiation and in maintaining the functional identity of β-cells [33], we focused on its putative regulation of miR-184 gene transcription. To investigate whether NKX6.1 indeed regulates miR-184 expression, we measured its expression in pancreatic islets isolated from 10 non-diabetic and 6 T2D donors (Supplementary Table 1). The results showed a significant reduction of NKX6.1 in T2D compared to non-diabetic pancreatic islets (Fig. 5b), which is consistent with the reported downregulation of miR-184-3p. As shown in Fig. 5c, the expression of NKX6.1 correlates positively with miR-184-3p levels. To investigate the role of NKX6.1 in miR-184 transcription, we silenced it in EndoC-βH1 cells (Fig. 5d, e and Supplementary Material Original WB) and then measured miR-184-3p expression; the results showed a significant decrease in miR-184-3p after NKX6.1 siRNA transfection (Fig. 5f).

Next, we wanted to determine whether NKX6.1 also plays a role in miR-184-3p expression in mouse β-cells. Therefore, we investigated whether translocation of NKX6.1 from the nucleus to the cytoplasm could be sufficient to cause a decrease in miR-184-3p transcription. Indeed, it was previously demonstrated that mouse β-cells exposed to oxidative stress showed translocation of NKX6.1 from the nucleus to the cytoplasm as a mechanism contributing to β-cell dysfunction [34]. Therefore, we exposed MIN6 cells to hydrogen peroxide (H_2_O_2_) and subsequently examined NKX6.1 translocation and miR-184-3p expression. In untreated MIN6 cells, NKX6.1 was mainly present in the nucleus, indicative of its functional and transcriptional activity (Fig. 5g, A–D). After H_2_O_2_ treatment, NKX6.1 partially translocated to the cytoplasm, as shown by immunofluorescence staining (Fig. 5g, E–H) and by NKX6.1-nucleus colocalisation analysis (Fig. 5h). Interestingly, the translocation of NKX6.1 from the nucleus to the cytoplasm was accompanied by a significant decrease in miR-184-3p (Fig. 5i) as well as MafA expression (Supplementary Fig. 9), a known NKX6.1 target gene [33, 35].

### NKX6.1 directly binds to miR-184 promoter sequence in human and in murine β-cells

**Fig. 6:**
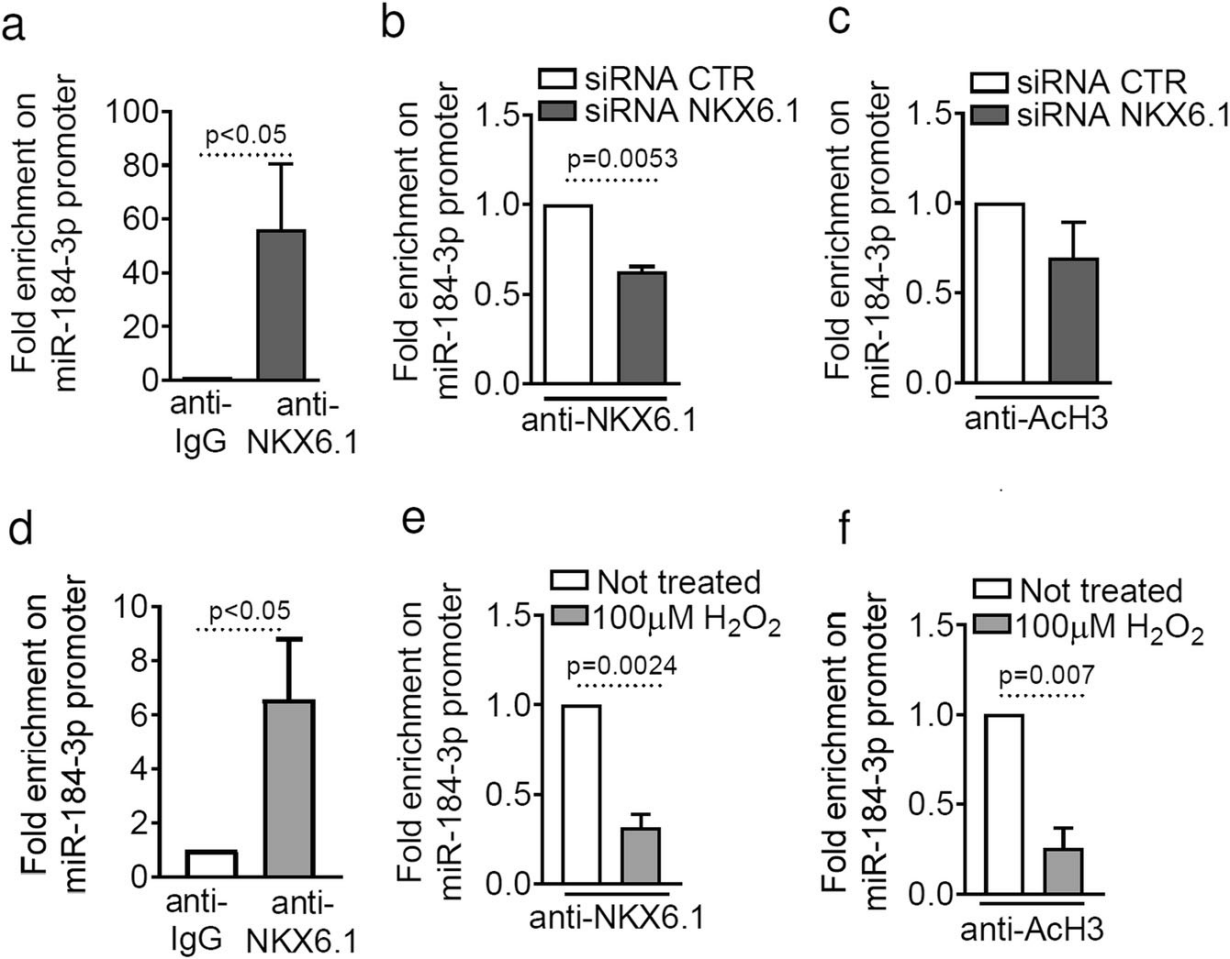
NKX6.1 binds directly to the miR-184 promoter. **a**–**c** ChIP-qPCR of NKX6.1 binding to the human miR-184 promoter sequence; data are shown as fold change in miR-184 promoter sequence bound by anti-NKX6.1 antibody compared to isotypic IgG negative control (**a**). ChIP-qPCR of NKX6.1 binding (**b**) or histone H3 acetylation (AcH3) (**c**) at the miR-184 promoter, with or without NKX6.1 siRNA and normalised to IgG control; values are shown as fold change in EndoC-βH1 cells transfected with NKX6.1 siRNA compared to scrambled siRNA; statistics using Mann–Whitney *U* test (*n* = 4–5 independent experiments). **d**–**f** ChIP-qPCR of NKX6.1 binding to the selected sequence of the murine miR-184 promoter; data are shown as fold change of anti-NKX6.1 antibody compared to isotypic negative control IgG (**d**); statistics using Mann–Whitney *U* test (*n* = 4 independent experiments). ChIP-qPCR of NKX6.1 binding (**e**) or histone H3 acetylation (AcH3) (**f**) at the murine miR-184 promoter, with or without 100 μM H_2_O_2_ treatment in MIN6 cells; values are shown as fold change of 100 μM H_2_O_2_ treated samples compared to untreated samples; statistics using Mann–Whitney *U* test (*n* = 4 independent experiments).

To distinguish between direct and indirect regulation of NKX6.1 on the miR-184 promoter, we performed chromatin immunoprecipitation (ChIP) analysis of the proximal regulatory region of the human MIR184 promoter, which contains NKX6.1 core binding sites. NKX6.1 binds efficiently to the putative selected sequence region of the MIR184 promoter in EndoC-βH1 cells (Fig. 6a). Accordingly, binding was significantly reduced after NKX6.1 siRNA transfection compared to scrambled siRNA control (Fig. 6b). Furthermore, immunoprecipitated chromatin associated with the miR-184-3p promoter sequence showed reduced acetylation on H3K9 and H3K14, indicating a lower site-specific chromatin activation state in the absence of NKX6.1 (Fig. 6c).

Finally, ChIP-qPCR analysis showed that NKX6.1 also binds to the regulatory promoter region of MIR184 transcriptional start site (TSS) in MIN6 cells (Fig. 6d–f). Notably, oxidative stress-induced translocation of NKX6.1 from the nucleus to the cytoplasm (Fig. 5g–h) reduced NKX6.1 binding to the MIR184 promoter region (Fig. 6e) and reduced acetylation of H3K9 and H3K14 (Fig. 6f) as a consequence of a decreased chromatin activation state.

Collectively, these data demonstrate that NKX6.1 is part of the transcriptional machinery that regulates miR-184-3p expression levels in both human and murine β-cells, thus linking the observed reduction in miR-184-3p to NKX6.1 expression and subcellular localisation in T2D.

## Discussion

The present study provides the first evidence for an NKX6.1-driven expression mechanism of miR-184-3p that plays a crucial role in T2D pathophysiology. The function of miR-184-3p was partially investigated in previous studies showing a possible role of this miRNA in β-cell physiology and T2D pathogenesis. Specifically, (i) miR-184-3p was shown to be one of the miRNAs enriched in β-cells/pancreatic islets [22]; (ii) it regulates insulin secretion by repressing the expression of SLC25A22 gene [25]; (iii) it regulates compensatory β-cell expansion by targeting AGO2 [22] and (iv) it is partially regulated by the energy sensor AMPK [26]. Despite this wealth of information, none of these studies has thoroughly characterised the molecular factors mediating the previously observed anti-apoptotic mechanism downstream of its reduction; furthermore, there is limited evidence for upstream regulators of miR-184-3p expression.

Using downstream and upstream bioinformatic approaches and subsequent analysis of T2D and non-diabetic human pancreatic islets as well as human (EndoC-βH1 and 1.1B4) and mouse β-cell lines (MIN6), we have here identified a direct target of miR-184-3p, as well as a specific β-cell transcription factor that mediates its transcriptional regulation. Specifically, we have identified and validated CRTC1 as a downstream target of miR-184-3p. CRTC1 is a member of the family of cAMP-regulated transcriptional coactivators (CRTCs) that binds to the bZIP domain of transcription factor CREB, resulting in increased occupancy of DNA CRE-responsive elements, thereby activating the expression of a specific gene pattern [36–38]. Indeed, CRTC1 is required for the efficient induction of CREB target genes. Moreover, CRTC1 activates CRE-responsive elements even in the absence of extracellular signalling, making CRTC1 a major modulator of CREB activity [39]. We have shown here that overexpression of CRTC1 in β-cells directly protects against lipotoxic- and cytokine-mediated apoptosis (IL-1β+TNF-α+IFN-γ), suggesting that this protective process is an important survival mechanism in β-cells. Interestingly, the protection mediated by miR-184-3p in human β-cells is completely dependent on CRTC1 expression, as it is completely abolished when CRTC1 is silenced. These results are consistent with the previously observed role of the CREB-CRTC complex. Indeed, transgenic mice expressing a defective form of CREB (A-CREB) developed hyperglycaemia due to an increased rate of the apoptosis of β-cells [40–42]. Therefore, it is conceivable that reduced or increased expression of the transcriptional coactivator CRTC1, which modulates CREB activity and thus the expression of its target genes, may contribute to the induction of or protection against apoptosis. Remarkably, this model fits the mechanism described by Martinez-Sanchez et al. who observed a link between AMPK-miR-184-3p and β-cell survival [26], finding that a reduction of AMPK activity induced by high glucose leads to a downregulation of miR-184-3p expression [43]. We can speculate that this phenomenon is related to a high glucose-dependent increase in cAMP, which in turn triggers further activation of CRTC1 [5, 44], whose expression is enhanced by inhibition of miR-184-3p.

While the protective effect of CRTC1 has been shown to be conserved between human and mouse β-cells, the mechanism leading to its overexpression, involving miR-184-3p, does not appear to be conserved. Indeed, CRTC1 is not a predicted target of miR-184-3p in mice and its expression was not modulated by inhibition of miR-184-3p (data not shown). However, the protective function exerted by downregulation of miR-184-3p was observed in both humans and mice. We can speculate that other mechanisms besides CRTC1 may mediate the protective effect in consequence of the decrease of miR-184-3p.

Another open question concerns the mechanism by which overexpression of CRTC1 protects β-cells from apoptosis; the pattern of downstream activated target genes involved in this process needs further investigation. However, a possible role in protection against apoptosis may be played by the miR-212/miR-132 axis, whose expression in β-cells has already been shown to be controlled by the CRTC1-CREB complex [45] and whose target genes are involved in protection against apoptosis in several cell types, including β-cells [46, 47].

Taken together, these results suggest the existence of a potential protective mechanism in β-cells, exerted by the miR-184-3p-CRTC1 axis leading to protection against apoptosis. This view is consistent with recent evidence suggesting that β-cell death contributes to the reduction of functional β-cell mass to a lesser extent than previously thought and that other mechanisms are involved in the loss of function of β-cells [4, 5, 48]. Our study provides evidence that NKX6.1 is downregulated in pancreatic islets from T2D donors and, more importantly, that it regulates miR-184-3p expression in both human and mouse β-cells by specifically binding to DRE present in its proximal promoter. NKX6.1 has been shown to be a β-cell-enriched transcription factor that controls a specific gene network required for β-cell function [34]; in addition, NKX6.1 maintains phenotype identity by controlling several β-cell-specific traits [35, 49]. Of importance, previous studies have also attributed a critical role in β-cell dedifferentiation to NKX6.1. Indeed, several lines of evidence suggest that: (i) NKX6.1 translocates from the nucleus to the cytoplasm in β-cells from T2D donors and *db/db* mice and was accompanied by activation of aldehyde dehydrogenase-1 a3 (Aldh1a3) and concomitant activation of typical undifferentiated endocrine cell marker genes [6, 50]; (ii) loss of function of NKX6.1 has been associated with the loss of β-cell-specific features, leading to disruption of some specific gene regulatory networks and consequently loss of β-cell identity [22]. Our data confirm the decreased expression of NKX6.1 in the pancreatic islets of T2D donors compared to non-diabetic controls. Furthermore, the expression of NKX6.1 in the islets was positively correlated with the expression of miR-184-3p, which may be due to direct transcriptional regulation. Although further studies are required to investigate the exact contribution of decreased expression of NKX6.1 and/or its translocation from the nucleus to the cytoplasm, to the expression of miR-184-3p, we can speculate that these processes may occur under stress conditions during T2D [6], and could lead to downregulation of miR-184-3p [5, 6]. The direct link between loss of function of NKX6.1 and dedifferentiation of β-cells has been demonstrated in several previous studies [5, 6], thus suggesting that the expression of miR-184-3p may be reduced as a consequence of the loss of function of NKX6.1 due to a dedifferentiation process, leading to upregulation of CRTC1 and protection of β-cells. Therefore, β-cells, although dysfunctional because of a dedifferentiation process, can be partially protected from apoptosis, indicating a possible link between dedifferentiation and protection. This model is supported by several recent findings linking the dedifferentiated phenotype of β-cells to protection against metabolic and inflammatory insults [7, 51, 52].

**Fig. 7:**
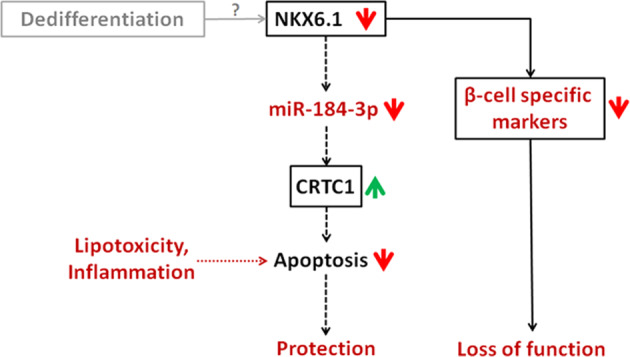
NKX6.1-miR-184-3p-CRTC1 axis. Working model of upstream and downstream miR-184-3p molecular mechanisms in β-cells protection.

In summary, we propose a model (Fig. 7) in which the observed reduction of miR-184-3p and consequent upregulation of its target gene CRTC1 in pancreatic islets from T2D donors follow the reduction/translocation of NKX6.1 observed in dedifferentiated and/or dysfunctional β-cells in T2D. This opens new potential avenues for therapeutic interventions aimed at protecting β-cells during diabetes.

## Materials and methods

### Human pancreatic islets

Human pancreatic islets were obtained from *n* = 13 non-diabetic (gender: 7 M, 6 F; age: 63.1 ± 11.9 years; BMI: 27.1 ± 3.8 kg/m^2^) and from *n* = 9 T2D multi-organ donors (gender: 6 M; 3 F; age: 72.2 ± 8 years; BMI: 26 ± 1.9 kg/m^2^) (see Supplementary Table 1). In detail, purified islets were isolated through injection of intraductal collagenase solution followed by density gradient purification as previously described [53]. Then, fresh human pancreatic islets preparations were handpicked, resuspended and maintained in culture in CMRL medium (cat. 11-530-037, Fisher Scientific, Pittsburgh, PA, USA) supplemented with L-Glutamine 1% (cat. G7513-100 mL), Antibiotic/Antimicotic 1% (A5955-100ML, Sigma-Aldrich, St. Louis, MO, USA), FBS 10%, in a 5% CO_2_ incubator at 28 °C. The experiments involving human participants were reviewed and approved by the local ethics committee of the University of Pisa (Italy). Islets were isolated from pancreata not suitable for organ transplantation, obtained following informed written consent by organ donors’ next-of-kin and with the approval of the local ethics committee.

### Cell culture and transfection

#### EndoC-βH1 cells

EndoC-βH1 human β-cell line [27, 54], obtained from UniverCell-Biosolutions (Toulouse-France), was used for all the experiments between passages 68–92. In detail, EndoC-βH1 cells were plated in coated flasks [coating medium: high glucose (4500 g/L) DMEM (cat. 51441 C), Penicillin/Streptomycin 1% (cat. P0781), ECM 1% (cat. E1270) and Fibronectin from bovine plasma 0.2% (cat. F1141)—all from Sigma-Aldrich, St. Louis, MO, USA] and maintained in culture in low glucose (1000 g/L) DMEM (cat. D6046) supplemented with 2% BSA fraction V (cat. 10775835001), β-Mercaptoethanol 50 µM (cat. M7522), L-Glutamine 1% (cat. G7513), Penicillin/Streptomycin 1% (cat. P0781), Nicotinamide 10 mM (cat. N0636), Transferrin 5.5 µg/mL (cat. T8158) and Sodium selenite 6.7 ng/mL (cat. S5261) - all from Sigma-Aldrich (St. Louis, MO, USA).

In order to evaluate CRTC1 mRNA and protein modulation by hsa-miR-184-3p, EndoC-βH1 cells were plated at a density of 1.37 × 10^5^ cells/cm [2] in 24- or 6-well plates. After 24 h, EndoC-βH1 cells were transfected with 100 nM of miRVANA miR-184 inhibitor (cat. 4464084, ID: MH10207- Invitrogen, Waltham, MA, USA) or with 100 nM negative control inhibitor (cat. 4464076- Invitrogen, Waltham, MA, USA) using Lipofectamine 2000 transfection reagent (cat. 11668-019, Invitrogen, Waltham, MA, USA).

NKX6.1 was silenced using specific siRNA as follows. EndoC-βH1 cells were plated at a density of 1.37 × 10^5^ cells/cm [2] in 24- or 6-well plates and were transfected after 24 h by using Lipofectamine 2000 transfection reagent (cat. 11668-019, Invitrogen, Waltham, MA, USA) for 48 h with 25 nM of CTR siRNA (cat. D-001810-10-05 ON-TARGET plus non-targeting pool siRNA 5 nmol, Dharmacon, Lafayette, CO, USA) or with 25 nM of NKX6.1 siRNA (cat. L-HUMAN-XX-0005, ID. L-020083-00-0005, ON-TARGET plus SMART pool 5 nmol, Dharmacon, Lafayette, CO, USA).

EndoC-βH1 cells were subjected to lipotoxic or inflammatory stimuli as previously described [55–57]. Briefly, lipotoxic stress was induced by 2 mM of sodium palmitate (cat. P9767-5G, Sigma-Aldrich, St. Louis, MO, USA) for 24 h or with EtOH 0.5% as a control treatment.

Pro-inflammatory stress was induced using a cytokines mix composed of IL-1β (50 U/mL) (cat. 201-LB-005, R&D system Minneapolis, MN, USA), TNFα (1000 U/mL) (cat. T7539 from Sigma-Aldrich, St. Louis, MO, USA) and IFNγ (1000 U/mL) (cat. 11040596001 from Roche, Basel, Switzerland) for 24 h.

To evaluate the role of CRTC1 and of hsa-miR-184-3p in β-cells protection and survival during lipotoxic (palmitate) or pro-inflammatory (cytokines mix) stress, EndoC-βH1 cells were plated at a density of 1.37 × 10^5^ cells/cm [2] in 24- or 6-well plates and transfected after 24 h using Lipofectamine 2000 transfection reagent (cat. 11668-019, Invitrogen, Pittsburgh, PA, USA) with different combinations of CRTC1 siRNA and/or miR-184 inhibitor: (i) 100 nM negative control miRNA inhibitor (cat. 4464076, Invitrogen, Pittsburgh, USA) + 50 nM of CTR siRNA (cat. D-001810-10-05 ON-TARGET plus Non-targeting Pool siRNA 5 nmol, Dharmacon- Lafayette, CO, USA); (ii) 100 nM negative control miRNA inhibitor + 50 nM of CRTC1 siRNA (cat. L-HUMAN-XX-0005, ID L-014026-01-0005, ON-TARGET plus SMART pool 5 nmol, Dharmacon- Lafayette, CO, USA); (iii) 100 nM of miRVANA miR-184 inhibitor (cat. 4464084, ID. MH10207-Invitrogen, Pittsburgh, MO, USA) + 50 nM of CTR siRNA; (iv) 100 nM of miRVANA miR-184 inhibitor (cat. 4464084, ID. MH10207-Invitrogen, Pittsburgh, USA) + 50 nM of CRTC1 siRNA. After 24 h, transfected EndoC-βH1 cells were subjected to lipotoxic or pro-inflammatory stress as reported above, and then analysed to evaluate pyknotic nuclei, cleaved Caspase-3 (FACS analysis of CASP3^+^ positive cells and western blot analysis) and evaluation of CRTC1 mRNA and protein expression.

#### MIN6 cells

MIN6 cells were obtained from AddexBio (San Diego, CA, USA), cultured between passages 11–25 and maintained in culture as previously described [58]. In detail, MIN6 were cultured in DMEM (cat. 5671, Sigma-Aldrich, St. Louis, MO, USA) supplemented with L-Glutamine 1%, Antibiotic/Antimicotic 1% (A5955, Sigma-Aldrich, St. Louis, MO, USA), FBS 15% (cat. ECS0180L, Euroclone, Milan, Italy), sodium pyruvate 1% (cat. ECM0542D, Euroclone, Milan, Italy) and β-Mercaptoethanol 50 µM. MIN6 were transfected with CRTC1 overexpressing plasmid (EX-Mm18750-Lv183) or with control plasmid (EX-EGFP-Lv151) (both from Genecopoeia, Rockville, MD, USA) through Lipofectamine 2000 transfection reagent. Lipotoxic stress was induced by using 0.5 mM of sodium palmitate (Sigma-Aldrich, St. Louis, MO, USA) or with EtOH 0.5% as a control treatment for 48 h as previously described [59]. Pro-inflammatory stress was induced by a cytokines mix composed of IL-1β (5 ng/mL, cat. I5271, Sigma-Aldrich, St. Louis, MO, USA), TNFα (30 ng/mL, cat. T7539) and IFNγ (10 ng/mL, cat. 485MI- R&D System, Minneapolis, MN, USA) for 24 h. Oxidative stress was induced using H_2_O_2_ (cat. H1009, Sigma-Aldrich, St. Louis, MO, USA) at 100 µM for 90 min.

#### 1.1B4 cells

1.1B4 human pancreatic β-cell line [60] (obtained from Sigma-Aldrich) was cultured in RPMI (cat. R0883, Sigma-Aldrich, St. Louis, MO USA) supplemented with L-Glutamine 1%, Antibiotic/Antimycotic 1%, FBS 10%. Transfection was performed by using Lipofectamine 2000 with 100 nM miRVANA miR-184 inhibitor (cat. 4464084, ID: MH10207-Invitrogen, Pittsburgh, PA, USA) with respect to 100 nM negative miRNA control inhibitor (cat. 4464076, Invitrogen, Pittsburgh, PA, USA).

#### HeLa cells

HeLa cells were cultured in DMEM (cat. 5671, Sigma-Aldrich, St. Louis, MO, USA) supplemented with L-Glutamine 1%, Antibiotic/Antimycotic 1%, FBS 10%. Lipofectamine LTX (cat. 15338100, Thermo Fisher Scientific, Waltham, MA, USA) was used for HeLa cell line transfection with plasmid DNA or with 100 nM of miRVANA miR-184 inhibitor or 100 nM negative control miRNA inhibitor (cat. 4464076, Invitrogen, Waltham, MA, USA).

### Predictive analysis of hsa-miR-184-3p target genes

TargetScan Human 7.0 (www.targetscan.org) algorithm was used to retrieve the list of conserved hsa-miR-184-3p predicted target genes (Supplementary Table 2).

### Dual-luciferase reporter assay

The luciferase reporter vector HmiT060400a-MT06, containing wild-type CRTC1 3′UTR sequence, and the vector HmiR0259-MR04, containing hsa-miR-184-3p precursor, were obtained from Genecopoeia (Rockville, MD, USA). HeLa cells were plated at a density of 4 × 10^4^/well in 24-well plates and, after 24 h, transiently co-transfected with 245 ng of hsa-miR-184-3p precursor plasmid vector and 5 ng of CRTC1 luciferase reporter vector, using 1.75 μL Lipofectamine LTX transfection reagent, for 48 h. After transfection, luciferase activity was measured at luminometer Glomax 20/20 using Dual-Luciferase Reporter Assay System (cat. #E1910-Promega, Fitchburg, USA) following the manufacturer’s instructions. The measurement of Firefly luciferase activity in frame with CRTC1 3’UTR sequence was normalised to Renilla luciferase one. Then, miRNeasy mini kit (Cat. 217004-Qiagen, Hilden, Germany) was used to extract total RNA at specific experimental points to assess the upregulation of miR-184-3p after transfection. Statistical significance was calculated using non-parametric unpaired two-tailed Mann–Whitney *U* test with a significance cutoff *p* < 0.05.

### qRT-PCR analysis

In order to evaluate miRNA and gene qRT-PCR expression analysis, total RNA (including small RNAs <200 nt) was extracted using miRNeasy mini kit (cat. 217004, Qiagen, Fitchburg, Germany). Then, total RNA concentration was assessed using Qubit 3000 Fluorometer (Thermo Fisher Scientific, Waltham, MA, USA), while RNA quality was evaluated using 2100 Bioanalyzer-RNA Pico Kit (Agilent Technologies), excluding those samples with RNA Integrity Number <5.0. For miRNA expression analysis, cDNA was prepared using TaqMan™ MiRNA Reverse Transcription Kit (cat. 4366596, Invitrogen, Waltham, MA, USA) according to the user manual, while cDNA from mRNAs was synthesised using SuperScript™ III First-Strand Synthesis System (cat. 18080044, Invitrogen, Waltham, MA, USA) alongside with random hexamers. Real-time PCR analysis was performed using Taqman miRNA expression assays or Taqman gene expression assays using the primers listed in Supplementary Table 3, following the manufacturer’s recommendations. Data were collected and analysed through Expression Suite software 1.0.1 (Thermo Fisher Scientific, Waltham, MA, USA) using 2^−ΔCt^ or 2^−ΔΔCt^ method. VIIA7 Real-time PCR instrument was used to analyse both miRNA and gene expression. Statistical significance was calculated using a non-parametric unpaired two-tailed Mann–Whitney *U* test (on 2^−ΔCt^) or paired parametric two-tailed t-test (on 2^−ΔΔCt^) with a significance cutoff *p* < 0.05.

### Gene Expression Omnibus (GEO) dataset analysis

GEO database (https://www.ncbi.nlm.nih.gov/gds) was interrogated to evaluate the expression of hsa-miR-184-3p predicted target genes in T2D pancreatic islets. We looked for previously published studies reporting gene expression profiling performed on pancreatic islets from T2D donors. We retrieved data from GSE20966 and GSE25724 datasets [28, 61] reporting the analysed and normalised results obtained from genes microarray profiling of pancreatic islets or LCM isolated β-cells from non-diabetic and T2D donors. Specifically, expression data corresponding to miR-184-3p predicted target genes were obtained and plotted by using GraphPad 8.0 software. Statistical significance was calculated using a non-parametric unpaired two-tailed Mann–Whitney *U* test with a significance cutoff *p* < 0.05.

### Western blot analysis

In order to evaluate CRTC1 expression, total proteins from EndoC-βH1, MIN6, 1.1B4 or HeLa cells were extracted using RIPA lysis buffer (50 mM Tris pH 7.6, 0.5% Sodium Deoxycholate, 1% IGEPAL, 0.1% SDS, 140 mM NaCl, 5 mM EDTA) supplemented with 1X protease inhibitors cocktail (Roche, Basel, Switzerland). Total proteins for cleaved Caspase-3 evaluation from EndoC-βH1 were extracted using RIPA lysis buffer (50 mM Tris-HCl pH 7.4, 150 mM NaCl, 1% Triton-X-100, 0.5% Sodium Deoxycholate, 0.1% SDS). Proteins were quantified using Bradford assay and 50 μg of proteins/lane were separated using Tris-Glycine 4–20% Mini-PROTEAN^®^ TGX™ Precast Protein Gels (Bio-Rad, Hercules, CA- USA). Proteins were then transferred to 0.2 μm PVDF membrane using a wet electrophoresis system. Upon transfer, PVDF membranes were washed three times with PBST1X 0.05% and then incubated 2 h with 5% non-fat dry milk in PBST1X 0.05%. To identify CRTC1, rabbit monoclonal anti-CRTC1 (cat. ab92477, Abcam) was diluted 1:1000 in 5% non-fat dry milk in PBST1X 0.05% and incubated o/n at 4 °C and then with Goat anti-rabbit (cat. SC-2004, Santa Cruz, Dallas, TX, USA) diluted 1:5000 in 2% non-fat dry milk in PBST1X 0.05% 1 h RT. NKX6.1 was detected using rabbit monoclonal anti-NKX6.1 (cat. 54551, Cell Signaling Technology, Danvers, MA, USA) diluted 1:500 in 5% non-fat dry milk in PBST1X 0.05% and incubated o/n at +4 °C and then with Goat anti-rabbit-HRP (Santa Cruz, Dallas, TX, USA, SC-2004) diluted 1:5000 in 2% non-fat dry milk in PBST1X 0.05% 1 h at RT. Activated Caspase-3 was detected using rabbit anti-cleaved Caspase-3 (Asp175) (cat. 9661, Cell Signaling Technology, Danvers, MA, USA); 0.2 μm PVDF membranes were blocked with 5% non-fat dry milk in TBST1X 0.1% for 2 h. Then, rabbit anti-cleaved Caspase-3 was diluted 1:1000 in 5% non-fat dry milk in TBST1X 0.1% and incubated o/n at +4 °C. Goat anti-rabbit peroxidase AffiniPure F(ab’)_2_ Fragment (cat. 111-036-003, Jackson ImmunoResearch, West Grove, PA, USA) was diluted 1:5000 in non-fat dry milk 2% in TBST1X 0.1% and incubated 1 h at RT.

CRTC1, NKX6.1 and cleaved Caspase-3 were normalised using β-actin; mouse monoclonal anti-β-actin (cat. A5441, Sigma-Aldrich, St. Louis, MO, USA) was diluted 1:10000 in 5% non-fat dry milk in PBST1X 0.05% or TBST1X 0.1% and incubated 1 h at RT and subsequently with Goat anti-mouse-HRP (Santa Cruz, Dallas, TX, USA-A1303) diluted 1:1000 in 2% non-fat dry milk in PBST1X 0.05% or TBST1X 0.1% 30 min at RT.

Chemiluminescent signal was detected by using ECL solution (GE Healthcare, Little Chalfont, Buckinghamshire, UK). Chemiluminescent analysis of immunoblot results was performed by densitometric analysis using LAS4000 analyzer (GE Healthcare, Little Chalfont, Buckinghamshire, UK-RPN2232) and Image J software. Statistical significance was calculated using a paired parametric two-tailed *t*-test (on fold change) with a significance cutoff *p* < 0.05.

### CRTC1 targeted mass spectrometric (MS) analysis

In order to perform CRTC1 targeted MS analysis, EndoC-βH1 cells were prepared as previously described [62, 63]. Briefly, cells were lysed with RIPA buffer (see above) and protein lysate was quantified through BCA assay. Then, protein lysate was mixed with 400 µL of urea 8 M in Tris-HCl 100 nM pH 8.5 (UA), with the addition of 100 mM DTT. The mixture was loaded on a filter 10 K Pall, incubated 30 min RT and centrifuged at 13,800 × g for 30 min. Filter was then washed twice with 400 µL of UA and centrifuged at 13,800 × g for 30 min, then incubated with 100 µL of 50 mM of iodoacetamide solution in a thermo-mixer for 1 min 150 rpm and without mixing for 20 min, then centrifuged at 13,800 × g for 20 min. Filter was then washed twice with 400 µL of UA and centrifuged at 13,800 × g for 30 min, twice with 400 µL of 50 mM ammonium bicarbonate (AMBIC) and centrifuged twice at 13,800 x g for 30 min and for 20 min, respectively. Then 40 µL of 50 mM AMBIC were added to the filter together with trypsin (ratio trypsin/proteins 1:25) and incubated O/N at 37 °C. Then, the sample was transferred in a collecting tube and centrifuged at 13,800 × g for 10 min. Subsequently, 100 µL of 0.1% formic acid were added on the filter and centrifuged 13,800 × g 10 min. Finally, filter was discarded, and solution was desalted with OASIS cartridges according to the manufacturer’s instructions. Then, peptides were concentrated using SpeedVac and the sample was resuspended in a solution of 3% acetonitrile, 96.9% H_2_O and 0.1% formic acid. Analyses were performed on a Q-Exactive Plus mass spectrometer (Thermo Fisher Scientific, Waltham, MA, USA), equipped with electrospray ion source operating in positive ion mode. The instrument is coupled to a UHPLC Ultimate 3000 (Thermo Fisher Scientific, Waltham, MA, USA). The chromatographic analysis was performed on a column Acquity UPLC Waters CSH C18 130 Å (1 mm × 100 mm, 1.7 µm, Waters) using a linear gradient with 0.1% formic acid in water (phase A) and 0.1% formic acid in acetonitrile (phase B). The flow rate was maintained at 100 μL/min and the column oven temperature at 50 °C. Mass spectra were recorded in the mass to charge (m/z) range 200–2000 at a resolution of 35 K at m/z 200. The raw data obtained were analysed using the Biopharma Finder 2.1 software from Thermo Fisher Scientific.

The elaboration process consisted of the comparison between the peak list obtained “in silico” considering the expected aminoacid sequence of human CRTC1 protein (Uniprot ID: Q6UUV9), trypsin as digestion enzyme and eventual modifications (carbamidomethylation, oxidation, etc.).

Peptides with the highest reliability were manually chosen for CRTC1, β-ACT and CHGA proteins. Then, aminoacid sequences of peptides of interest were identified to collect m/z and NL values. To obtain fold change, NL values of CRTC1 were normalised for NL values of both β-ACT and CHGA. One-Way ANOVA with Tukey multiple comparison test was used for statistical analysis and a significance cutoff was set as *p* value < 0.05.

### Cytofluorimetric analysis and pyknotic nuclei count for apoptosis detection

Apoptosis rate was detected by cytofluorimetric analysis of cleaved Caspase-3 positive cells and by pyknotic nuclei count. The detection of cleaved Caspase-3 levels was performed on MIN6 and EndoC-βH1 cells fixed and permeabilized with BD Perm/Wash (cat. 554723-BD, Biosciences, Franklin Lakes, New Jersey, USA) and Cytofix/Cytoperm (cat. 554722-BD, Biosciences, Franklin Lakes, New Jersey, USA) buffers and stained by using 10 μg/mL of rabbit polyclonal anti-cleaved Caspase-3 (cat. ab13847, Abcam) antibody [or 10 μg/mL of rabbit IgG Isotype Control (cat. ab199376 Abcam)] and 1:500 diluted secondary antibody Goat anti-rabbit Alexa Fluor 594 (cat. A11037, Thermo Fisher Scientific, Waltham, MA, USA). Cells were analysed in a BD FACS Canto flow cytometer with FACS Diva software (Franklin Lakes, New Jersey). Then, FlowJo software was used to analyse cleaved Caspase-3-positive cells.

Pyknotic nuclei were counted using Hoechst 33342 (cat. 62249, Invitrogen, Waltham. MA, USA) diluted 1:100 and added to 24-well plates at 1 µL/well. Manual counting was used to determine the number of pyknotic nuclei (normalised to the number of total nuclei) in five different areas/well. Manual counting was performed using an inverted fluorescence microscope (Leica DMI3000 B, Leica Microsystems, Wetzlar, Germany) at ×40 magnification.

One-way ANOVA with Tukey multiple comparison test was used for statistical analysis and a significance cutoff was set as *p* value < 0.05.

### Predictive analysis of miR-184 promoter regulation

The MatInspector algorithm (https://www.genomatix.de/online_help/help_matinspector/matinspector_help.html) was used to identify transcription factors that are thought to have one or more recognition sites on the DNA sequence corresponding to the proximal promoter of miR-184, 500 bp upstream of the TSS. The sequence 500 bp upstream of miR-184 TSS, corresponding to the proximal promoter, as already described [31], was retrieved from the Ensembl database for the human (ENST00000384962) and murine sequence (ENSMUST00000083662), and selected to perform the bioinformatic analysis.

### NKX6.1 immunofluorescence analysis

MIN6 cells were immunostained as follows: treated or untreated cells were fixed in 4% PFA for 10 min, permeabilized in 0.25% Triton-X-100 for 5 min and blocked in 3% BSA + 0.05% Triton-X_100 in PBS for 30 min. MIN6 cells were incubated with the primary rabbit monoclonal antibody against NKX6.1 (cat. 54551, Cell Signaling Technology, Danvers, MA, USA) diluted 1:100 in BSA 1% in PBS for 1 h, rinsed with PBS and incubated with goat anti-rabbit Alexa Fluor-488 in 1% BSA in PBS (1:500), which was used as a secondary antibody.

### Chromatin immunoprecipitation (ChIP) analysis

ChIP analysis was performed using the MagnifyTM Chromatin Immunoprecipitation System (cat. 492024, Thermo Fisher Scientific, Waltham, MA, USA) according to the manufacturer’s instructions. The cultured MIN6 and EndoC-βH1 cell lines were incubated with 1% formaldehyde for 10 min. Fixation was stopped at room temperature with 114 µL of 1.25 M glycine and then samples were washed with PBS and 199 µL of lysis buffer in the presence of 1 µL of Protease Inhibitor 200X at 4 °C. Chromatin shearing was performed through sonication. The sonicated fragments were verified on a 2% TAE agarose gel. The sonicated chromatin was added to 0.2 mL PCR tube containing primary antibody [3 µg of rabbit anti-NKX6.1 (cat. 54551, Cell Signaling Technology, Danvers, MA, USA) or 3 μg of rabbit anti-acetyl-histone H3- cat. 06599, Millipore, St. Louis, MO, USA)], coupled to the protein A/G Dynabeads. The rabbit IgG isotype control antibody (cat. 02–6102, Invitrogen, Waltham, MA, USA) was used as negative control. Samples were incubated 2 h at 4 °C. After incubation, chromatin was washed with IP Buffer 1 and IP Buffer 2 provided by kit. After the last wash, Reverse Crosslinking Buffer + Proteinase K was added to all samples, followed by incubation at 55 °C for 15 min; chromatin immunoprecipitated with specific antibodies and non-immunoprecipitated chromatin (referred to as input control) was then recovered and incubated at 65 °C for 15 min. Samples were incubated with DNA Purification Buffer + DNA Purification Magnetic Beads and then washed with DNA Wash Buffer and DNA Elution Buffer. After incubation at 55 °C for 20 min, samples were stored at −20 °C.

### Real-time PCR for ChIP analysis of miR-184 promoter

Real-time PCR was performed to quantify the amplification of the immunoprecipitated miR-184-3p sequence promoter fragment. 1/30 of the recovered DNA was added to the PCR reaction, which consisted of 12.5 µL Maxima SYBR Green qPCR Master mix (2×), 0.05 µl ROX solution and nuclease-free H_2_O. The following primers were used for the human MIR184 gene promoter (300 nM each): forward primer 5’-AATGGCATGTGGGTGTTGGT-3’; reverse primer 5’-AGGGCTCCTGCAGGTCTGA-3’. The following primers were used for miR-184-3p murine promoter (500 nM): forward primer 5’-AATGGCATGTGGGTGTTGGT-3’; reverse primer 5’-AGGGCTCCTGCAGGTCTGA-3’.

The reaction was incubated at 95 °C for 10 min, 95 °C for 15 s – 60 °C for 30 s – 72 °C for 30 s (40 cycles) in ViiA7 Real-time PCR instrument (Thermo Fisher Scientific, Waltham, MA, USA). Data were collected and analysed with Expression Suite software 1.0.1 (Thermo Fisher Scientific, Waltham, MA, USA) using 2^−ΔCt^ method. The raw Ct data resulting from chromatin immunoprecipitated with specific antibodies were normalised to the negative isotypic control rabbit IgG (cat. 492024, Thermo Fisher Scientific, Waltham, MA, USA). Statistical significance was calculated using a paired parametric two-tailed *t*-test (on fold change) with a significance cutoff *p* < 0.05.

## Supplementary information

Supplementary Material

Supplementary Original Western Blot

Supplementary File 1

Supplementary File 2

## Data Availability

All data generated in this work have been included as primary and/or supplementary material and are also available on request from the corresponding author.
