# Supplementary material for: Reduced miR-184-3p expression protects pancreatic β-cells from lipotoxic and proinflammatory apoptosis in type 2 diabetes via CRTC1 upregulation
Source: Cell Death Discov. 2022 Jul 29;8:340. doi: 10.1038/s41420-022-01142-x (PMC9338237; doi:10.1038/s41420-022-01142-x)

Orginal Western Blots related to Figure 3d

Figure 3d

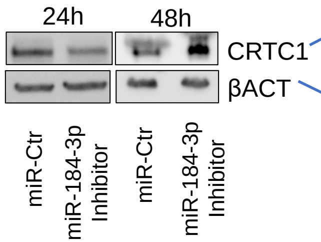

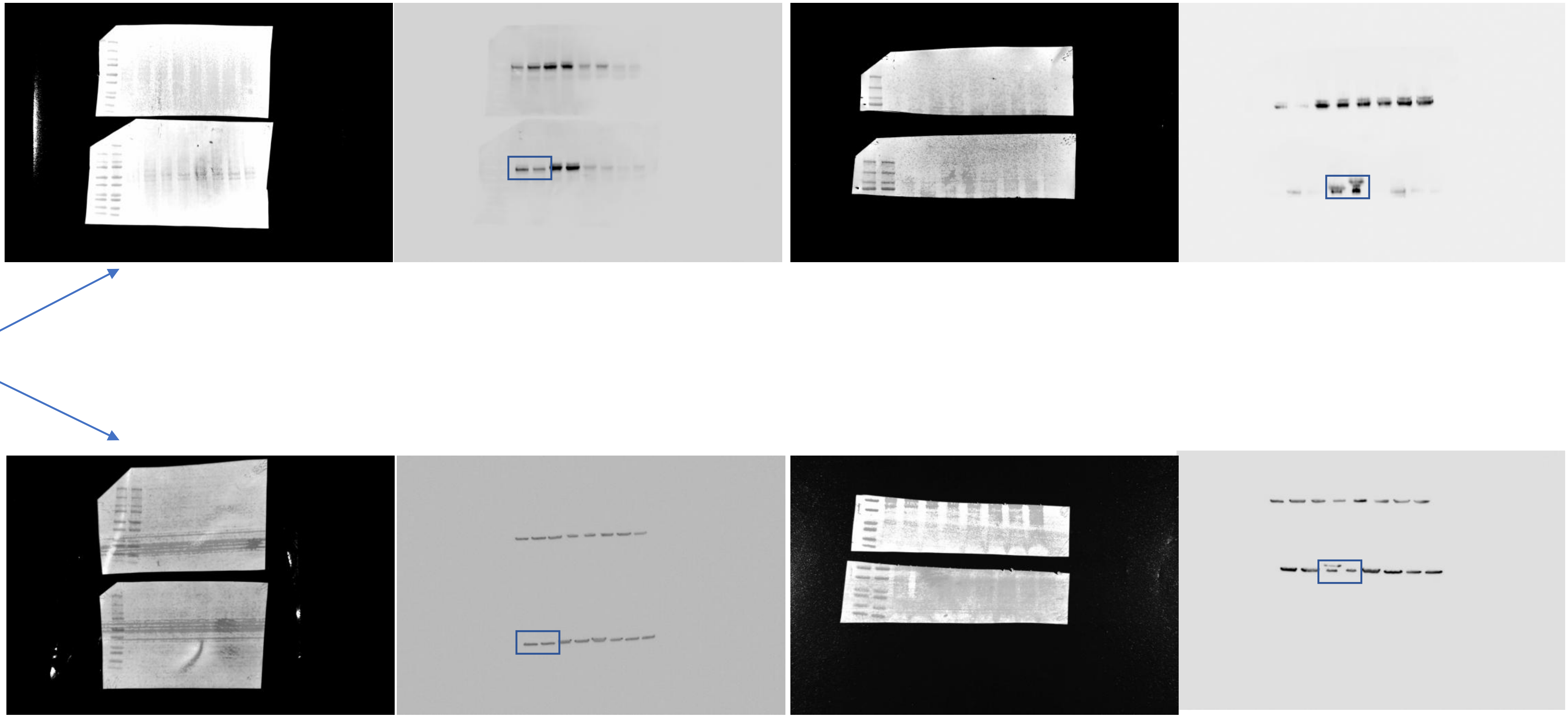

Orginal Western Blots related to Supplementary Figure 2b

Supplementary Figure 2b

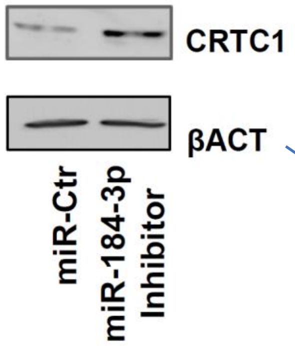

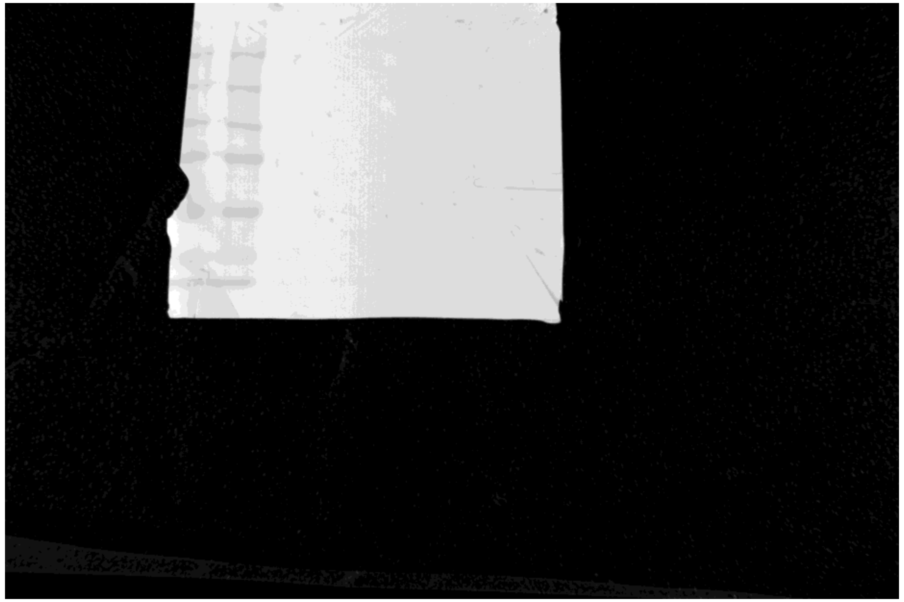

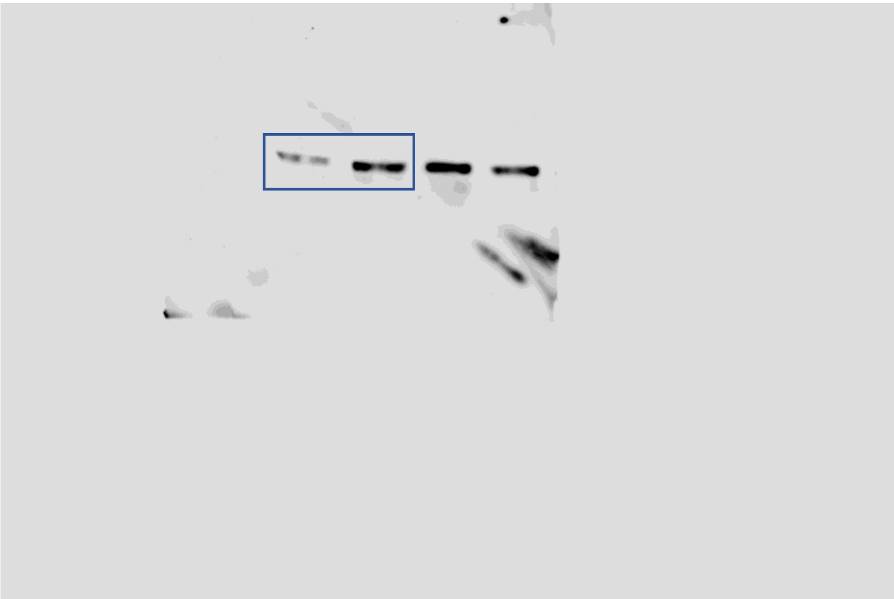

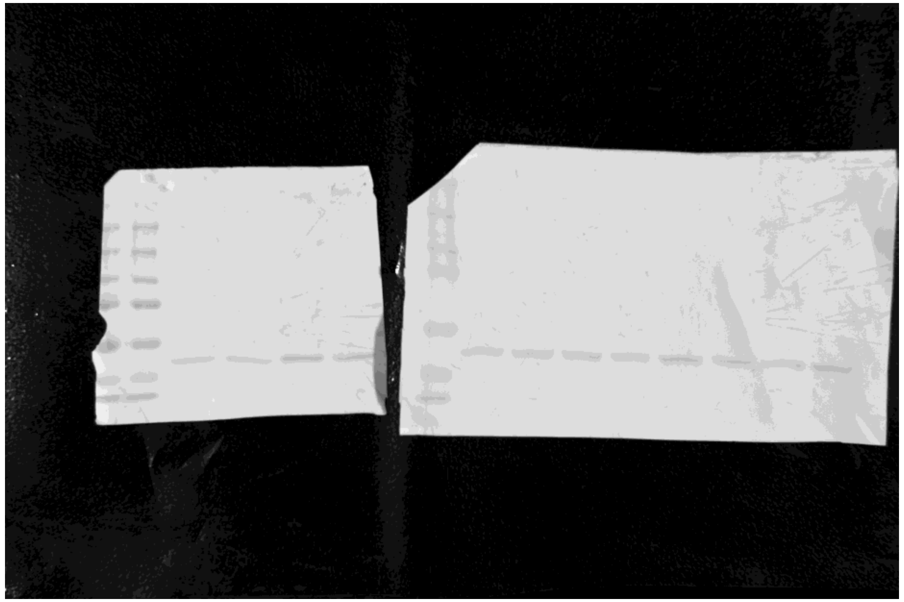

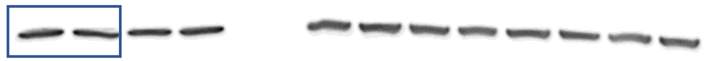

Orginal Western Blots related to Supplementary Figure 2d

Supplementary Figure 2d

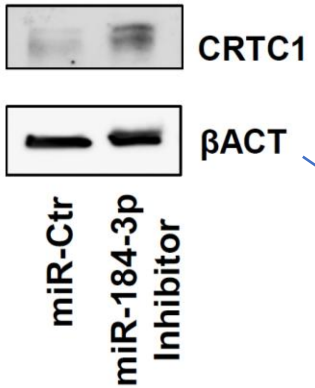

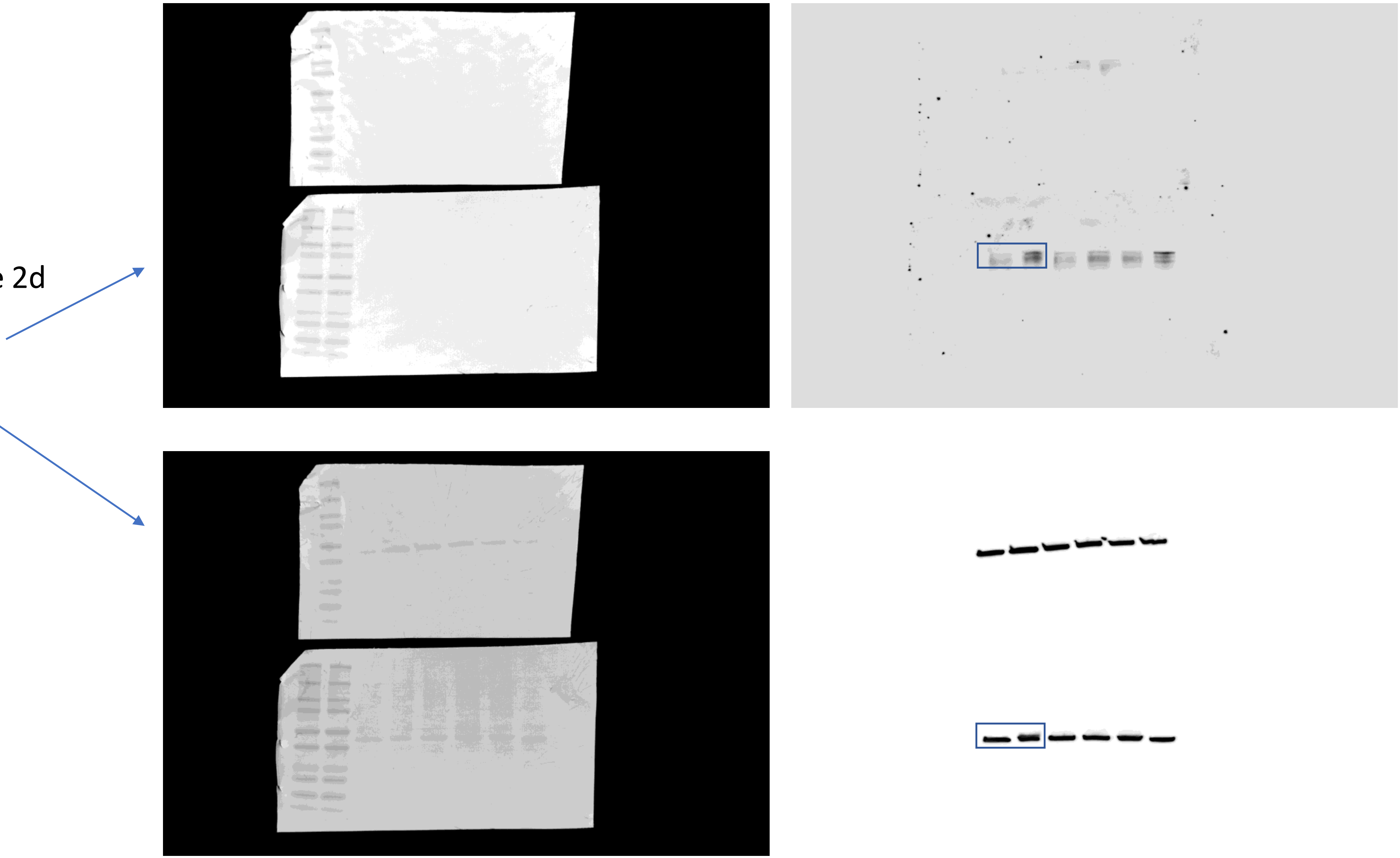

Orginal Western Blots Related to Figure 4e

Figure 4e

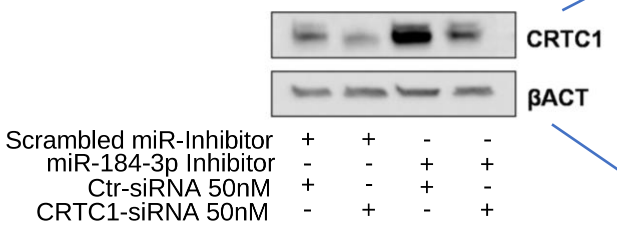

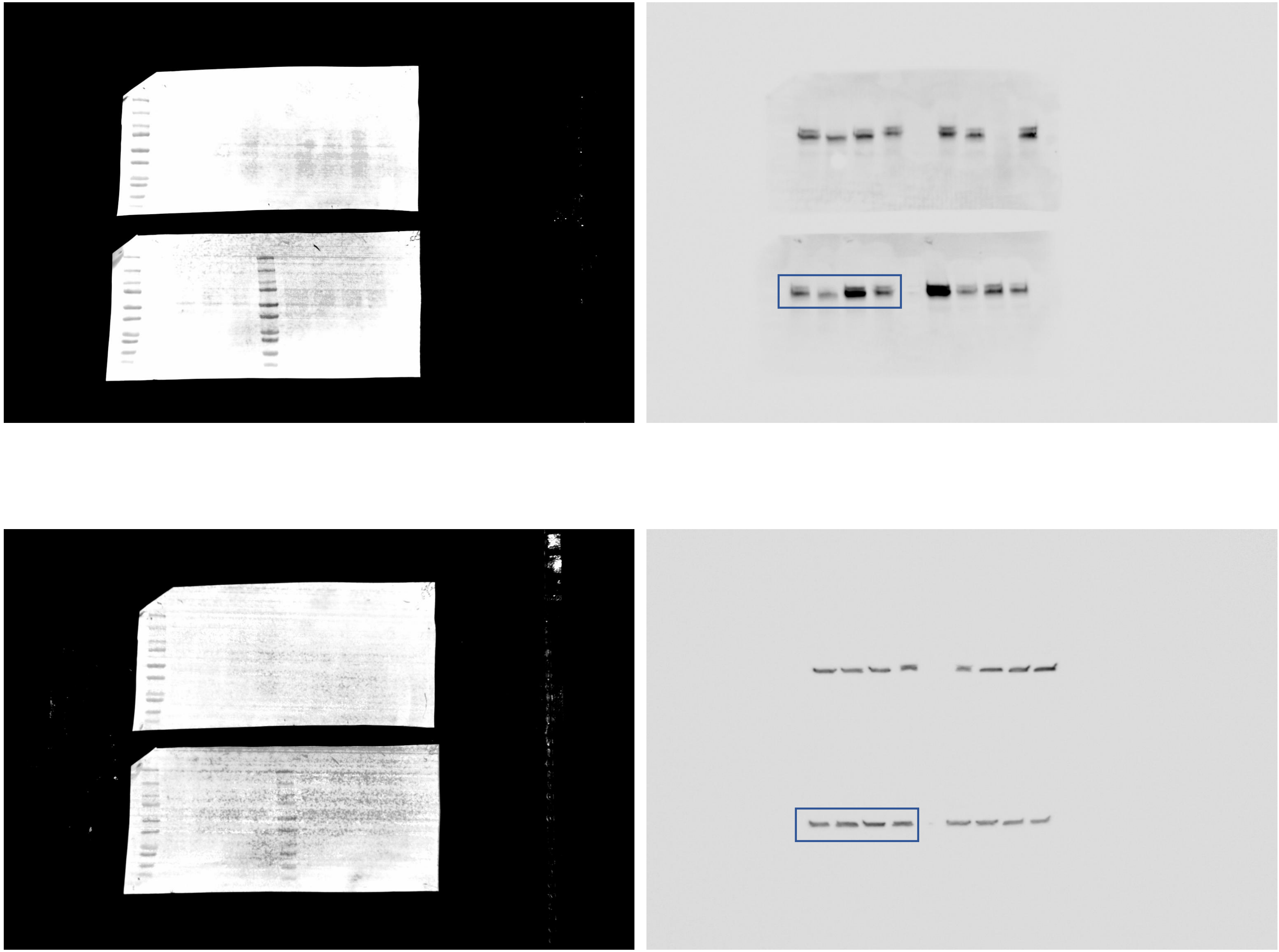

Orginal Western Blots related to Figure 4g

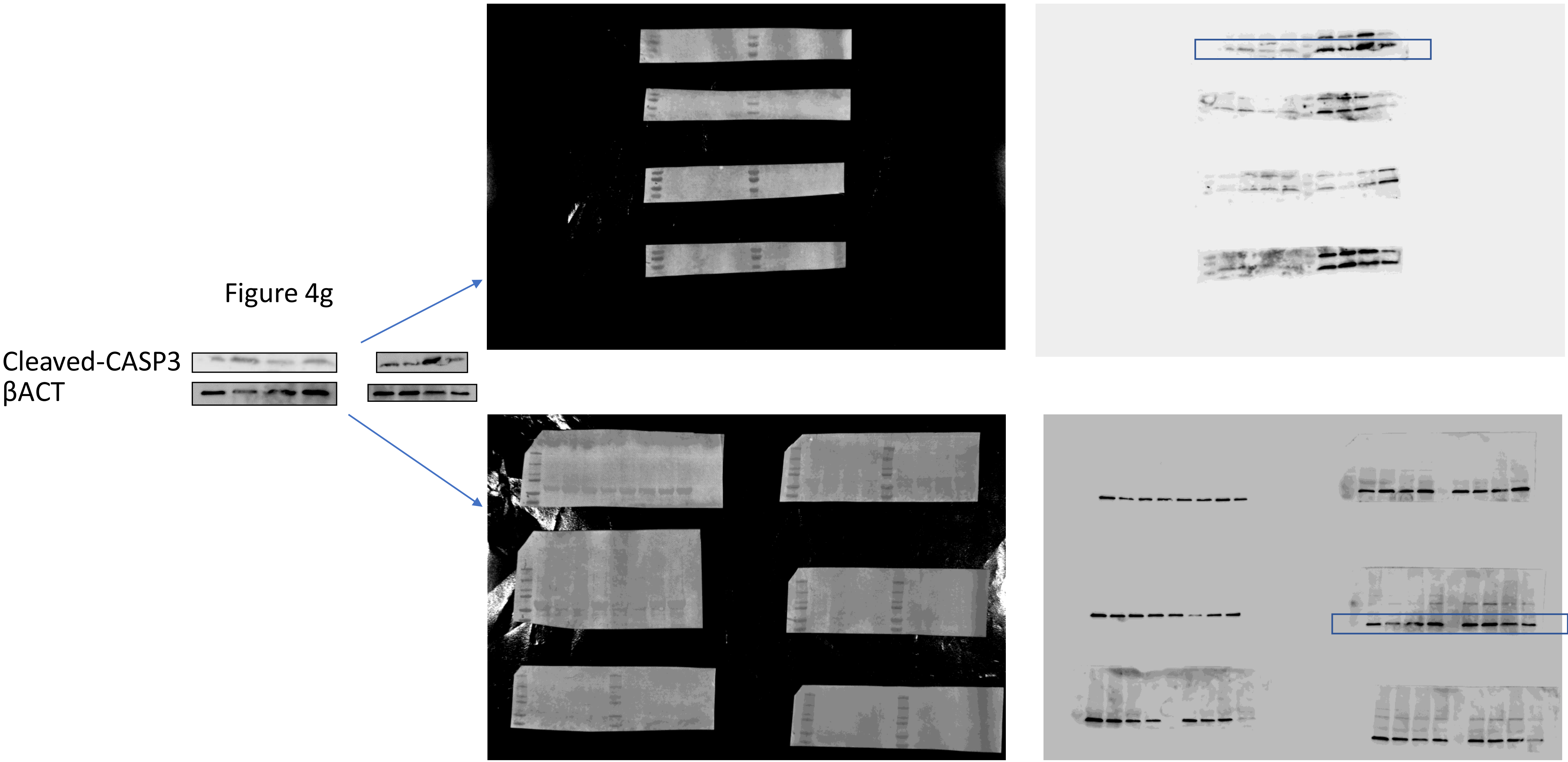

Orginal Western Blots related to Figure 4h

Figure 4h

Cleaved-CASP3
βACT

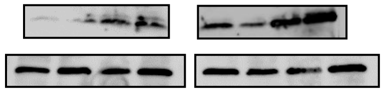

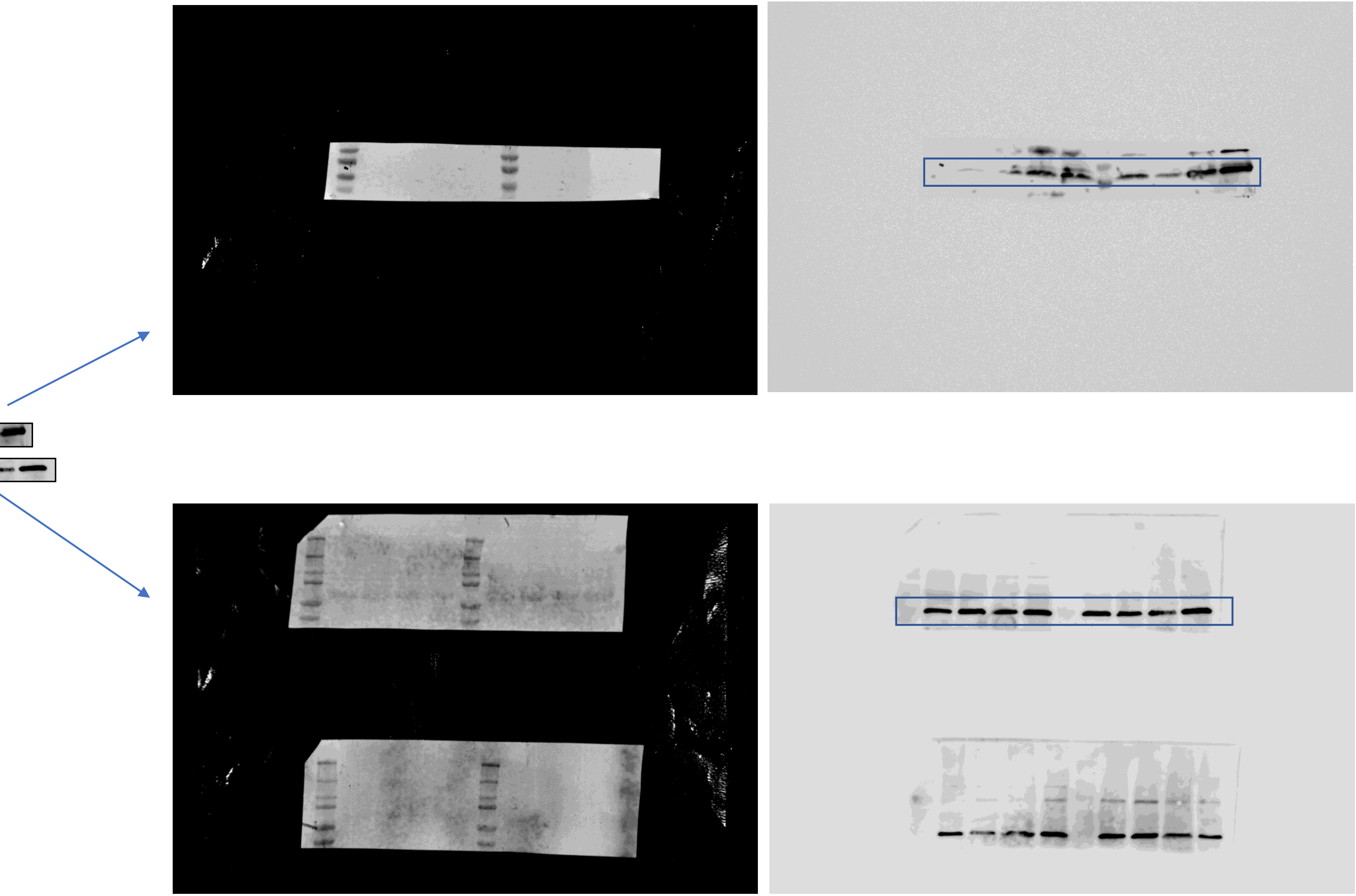

Orginal Western Blots related to Figure 5e

Figure 5e

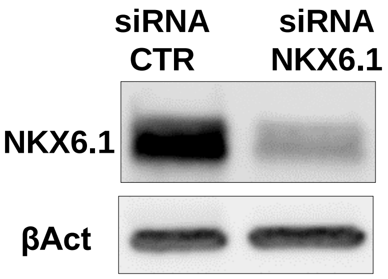

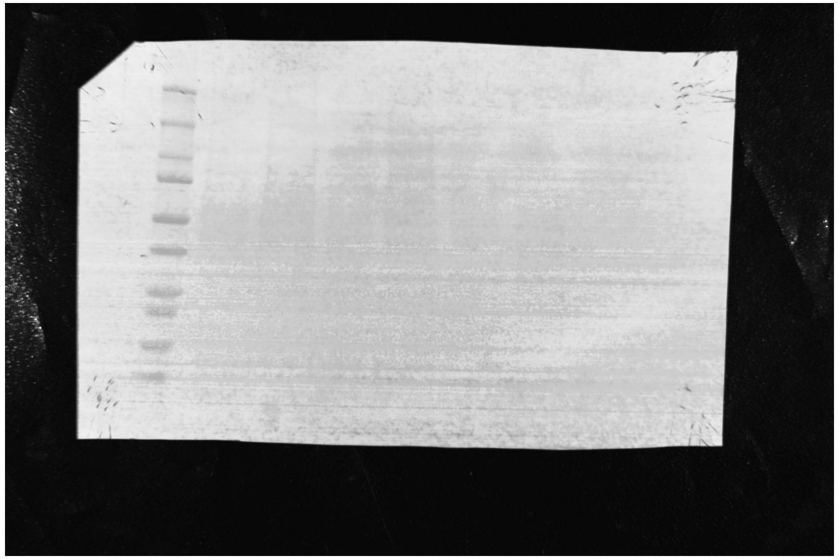

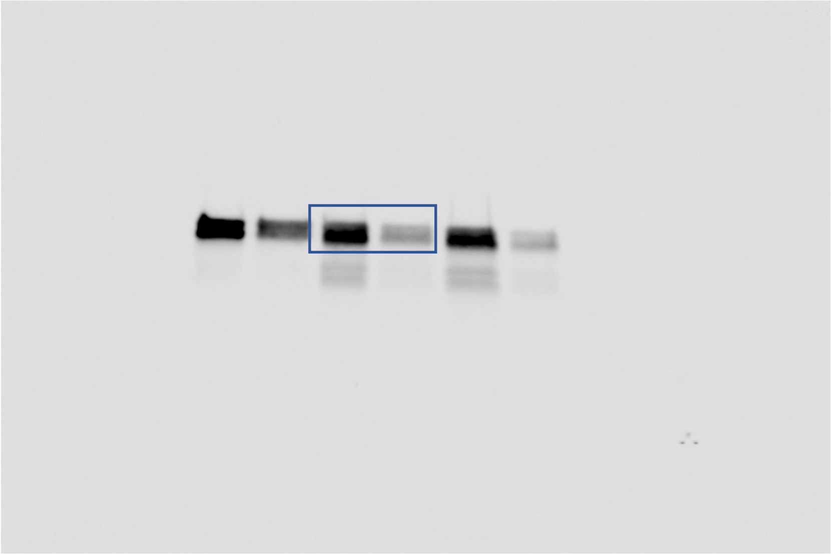

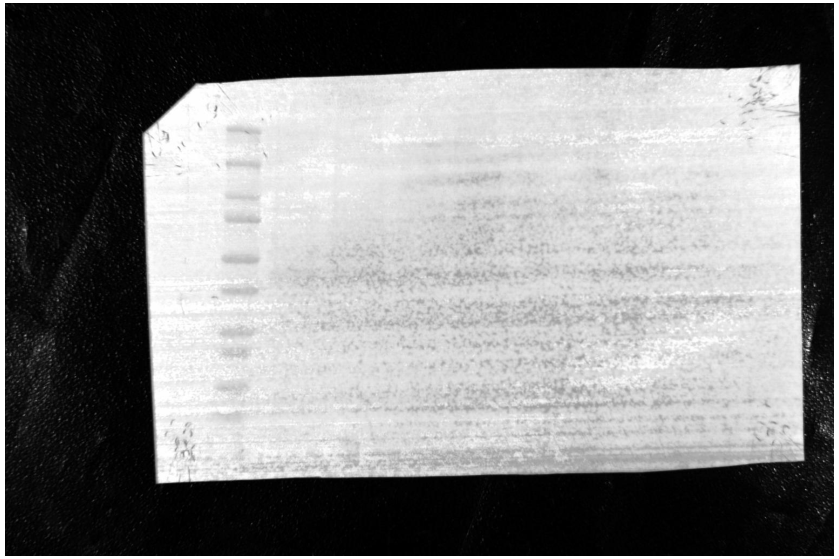

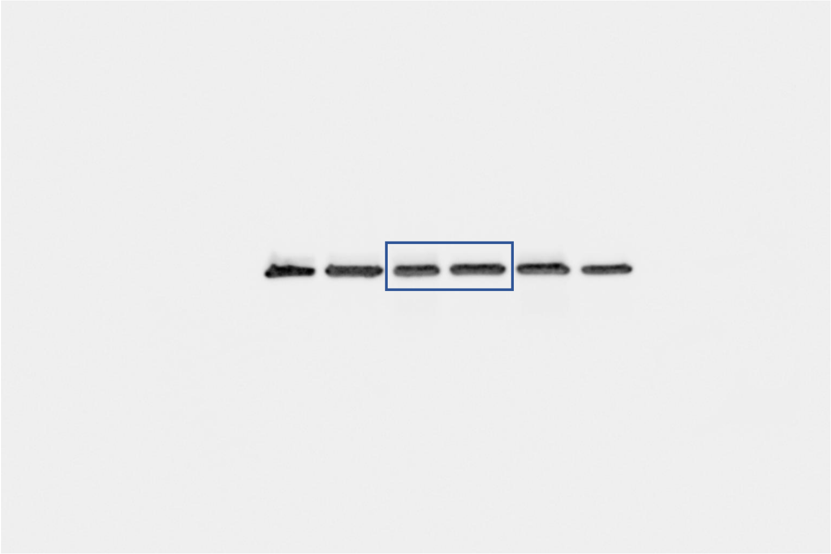

Supplement: Supplementary file 2 — Supplementary Original Western Blot [file 41420_2022_1142_MOESM2_ESM.pdf]
